# Functional Rescue of F508del-CFTR Using Small Molecule Correctors

**DOI:** 10.3389/fphar.2012.00160

**Published:** 2012-09-26

**Authors:** Steven Molinski, Paul D. W. Eckford, Stan Pasyk, Saumel Ahmadi, Stephanie Chin, Christine E. Bear

**Affiliations:** ^1^Programme in Molecular Structure and Function, Research Institute, Hospital for Sick ChildrenToronto, ON, Canada; ^2^Department of Biochemistry, University of TorontoToronto, ON, Canada; ^3^Department of Physiology, University of TorontoToronto, ON, Canada

**Keywords:** F508del-CFTR folding, trafficking, conformational stability, intra-molecular defects, small molecule correctors, drug discovery

## Abstract

High-throughput screens for small molecules that are effective in “correcting” the functional expression of F508del-CFTR have yielded several promising hits. Two such compounds are currently in clinical trial. Despite this success, it is clear that further advances will be required in order to restore 50% or greater of wild-type CFTR function to the airways of patients harboring the F508del-CFTR protein. Progress will be enhanced by our better understanding of the molecular and cellular defects caused by the F508del mutation, present in 90% of CF patients. The goal of this chapter is to review the current understanding of defects caused by F508del in the CFTR protein and in CFTR-mediated interactions important for its biosynthesis, trafficking, channel function, and stability at the cell surface. Finally, we will discuss the gaps in our knowledge regarding the mechanism of action of existing correctors, the unmet need to discover compounds which restore proper CFTR structure and function in CF affected tissues and new strategies for therapy development.

## Molecular Defect Caused by F508del in CFTR

The major Cystic Fibrosis mutation, F508del, causes multiple defects in the CFTR protein, leading to its impaired assembly during synthesis and reduced post-translational stability. Recently, it has been argued that a single small molecule compound may be unable to “correct” the conformational maturation, channel activity, and unfolding of the full-length mutant protein at the cell surface, given the existence of multiple intra- and inter-domain defects.

On the basis of biophysical studies of the isolated first nucleotide binding domain (NBD1) bearing the F508del mutation, together with biochemical studies of the full-length mutant protein, it has become clear that the deletion of F508 induces multiple structural defects in CFTR (Du et al., 2005; Serohijos et al., 2008; Du and Lukacs, 2009; Thibodeau et al., 2010; Yu et al., 2011; Aleksandrov et al., 2012; Mendoza et al., 2012; Rabeh et al., 2012). The absence of F508 in NBD1 leads to kinetic and thermal instability of the isolated domain (Protasevich et al., 2010; Wang et al., 2010a). Biochemical studies of the full-length protein in cell membranes have revealed that F508del-NBD1, in the amino terminal half of the protein, fails to mediate appropriate interactions with the carboxy terminal half of the protein (Du et al., 2005; Du and Lukacs, 2009). Both the intra-domain (NBD1) and the intra-molecular (CFTR) defects will be discussed in the following paragraphs. Furthermore, we will discuss recent evidence supporting the idea that both intra-domain and intra-molecular aberrations will need to be corrected in order to restore near Wt biosynthesis and post-translational stability to F508del-CFTR.

### NBD1: Intra-domain defects conferred by F508del

**Figure 1:**
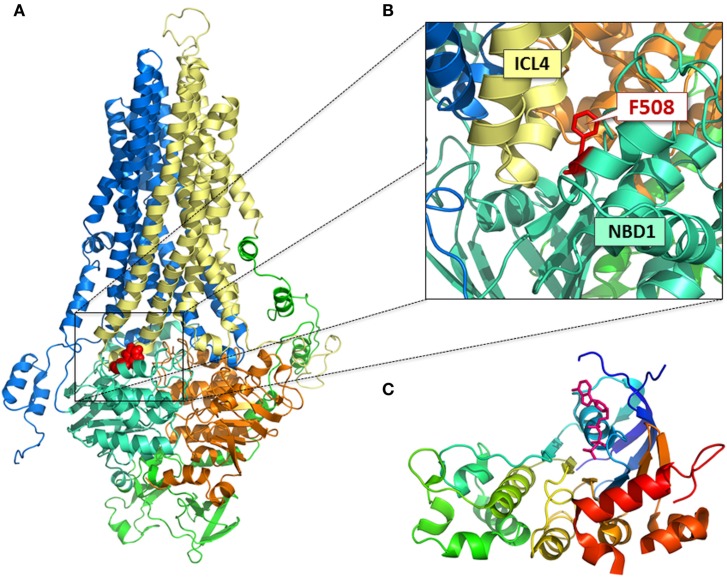
**Structural models of CFTR**. **(A)** Full-length homology model of CFTR (Mornon et al., 2009); MSD1, blue; MSD2, yellow; NBD1, cyan; NBD2, orange; R domain, green; F508, red; **(B)** position of F508 at the ICL4:NBD1 interface; **(C)** crystal structure of NBD1 (Lewis et al., 2010); ATP, pink.

The crystal structures of human NBD1 (Wt and F508del) were generated using proteins bearing second site mutations, introduced to confer stable protein fragments suitable for such structural studies (Lewis et al., 2004, 2005, 2010). As a result, these models lack information regarding the relative thermodynamic instability of the mutant protein. However, the crystal structures do provide a structural template with which to compare CFTR nucleotide binding domains (NBDs) with the NBDs of other ABC family members. As for other family members, NBD1 of CFTR possess a central, core F1-ATPase like subdomain, comprised of two non-contiguous sequences, i.e., the amino terminal region: G451-P499 and the carboxy terminal region (including D565-Q637). The NBD1 of CFTR also possesses an α-helical subdomain (495–565), conserved amongst other members of the ABC superfamily of transporters. F508 resides in this α-helical subdomain. Unique features of CFTR-NBD1 include disordered or flexible regions that are either missing or exhibit multiple orientations in the crystal structure. These unique regions include the “regulatory insertion” (RI: 405–436), the “structurally diverse region” (SDR: 536–550), and the “regulatory extension” (RE: 656–673). This latter region is now considered to comprise the amino terminal region of the phosphorylated, regulatory region, called the “R domain.” As shown in Figure 1, the amino and carboxy terminal residues of NBD1 are close to one another, underscoring the complexity of its folding involving the formation of specific subdomain interactions.

Biophysical studies of F508del-NBD1 in solution (some lacking the so-called, second site “stabilizing” mutations) revealed inherent alterations in kinetic and thermal stability (Protasevich et al., 2010; Wang et al., 2010a). In isothermal denaturation studies, Hunt and colleagues showed that NBD1 unfolding is strongly influenced by F508del and that unfolding is delayed by the “stabilizing” mutations utilized in the crystal studies (Wang et al., 2010a). Similarly, Brouillette and colleagues showed that F508del also influenced temperature dependent unfolding of NBD1 (Protasevich et al., 2010). In both cases, Mg-ATP binding delays unfolding of the Wt but not the mutant NBD1. The FRET-based folding studies by the Skatch group showed that while F508del mutation does not impair ATP binding, it does impair ATP-dependent interactions between the two non-contiguous regions (amino and carboxy terminal regions) the core F1-ATPase domain. These studies highlight the potential consequences of F508del on the canonical (ABC protein) subdomain interactions in NBD1.

Other biophysical studies highlight the potential consequences of F508del on the interactions mediated by the non-conserved or unique regions of CFTR. NMR studies of F508del-NBD1 (bearing certain stabilizing mutations) revealed alterations in the phosphorylation-regulated affinity of flexible regions (specifically the RI region) with the core of the NBD1 domain (Kanelis et al., 2010). Interestingly, this intra-domain interaction is strengthened in F508del, leading the authors of this work to speculate that F508del causes allosteric changes in NBD1 affecting not only intra-domain interactions but possibly, also preventing critical intra-molecular interactions as well.

As previously mentioned, second site “stabilizing mutations” protect the isolated F508del-NBD1 from unfolding in denaturation studies. The first stabilizing mutations were identified in the ABC conserved, canonical subdomains, and cluster in the α-helical subdomain (G550R, R553Q, R555K), in the γ switch (F494N), and ATP binding core subdomain (Q637R). These findings support the claim that interactions between these canonical subdomains are perturbed by F508del and more importantly, that these regions could constitute targets for pharmacological intervention. More recently, it was determined that substitution of residues in the (SDR: 536–550) of human F508del-NBD1 to residues found in avian F508del-NBD1 led to a profound increase in biosynthetic maturation of the full-length protein (Aleksandrov et al., 2012). These findings together with previous studies of the RI region (Aleksandrov et al., 2010), prompt the speculation that these dynamic, disordered regions of F508del-CFTR are important in mediating intra-molecular as well as intra-domain folding.

### Intra-molecular defects conferred in CFTR by F508del

It has been shown that intra-domain defects caused by F508del in NBD1 lead to defects in assembly of the full-length protein and to defects in post-translational stability. It is well known that defective assembly of F508del-CFTR is detectable as the marked reduction in the conversion of core glycosylated F508del-CFTR to complex glycosylated protein and decreased functional expression on the cell surface (Cheng et al., 1990). This hallmark biochemical profile (i.e., reduced complex glycosylated protein on a Western blot) reports the ER retention of the mutant protein. Lukacs and colleagues were the first to probe the conformational defects of the mutant protein using limited proteolysis (Du et al., 2005; Du and Lukacs, 2009). Protease resistance is known to provide insight into the conformational compactness of proteins folded in cells and the protease resistance of F508del-CFTR was shown to be significantly reduced relative to the Wt-CFTR protein. Protease digest patterns, analyzed by SDS-PAGE and probed using domain specific antibodies, revealed that the protease resistance of NBD2 was particularly reduced in the context of the full-length mutant protein, relative to the full-length Wt-CFTR. These were the first data to reveal the possible consequences of a misfolded F508del-NBD1 on assembly with the second half of the CFTR protein during translation.

Misassembly of the full-length F508del-CFTR protein likely occurs at several intra-molecular junctures as there are multiple loci at which NBD1 directly interacts with domains in the second half of the full-length protein. The identification of a pivotal juncture was guided by molecular models of the full-length CFTR protein generated using the crystal structure of the bacterial ABC transporter, Sav1866 as a template (Figure 1). In the models of CFTR based on Sav1866 protein, NBD1 interacts with NBD2 and with MSD2. NBD1 interacts with MSD2 via the coupling helix presented by the long helical extension known as intracellular loop 4 (ICL4; Figure 1B; Serohijos et al., 2008; Mornon et al., 2009; Dalton et al., 2012). The consequence of F508del in disrupting the NBD1: NBD2 interface is still under investigation. To date, interventions aimed at disrupting or enhancing this interaction do not appear to affect biosynthesis and processing of F508del-CFTR (Thibodeau et al., 2010). These findings suggest that, even if F508del-CFTR is shown to perturb this interface, this would not have a significant effect on CFTR folding. On the other hand, interventions aimed at modifying the interaction between the surface on NBD1 lacking F508 and the coupling helix presented by ICL4 significantly enhance the biosynthesis and processing of F508del-CFTR (Mendoza et al., 2012; Rabeh et al., 2012).

Disease-causing mutations in the coupling helix of ICL4 that cause ER retention have been described (L1065P, R1066C, and G1069R), supporting the idea that this region mediates important interactions during folding (Mendoza et al., 2012). Substitution of the arginine at position 1070 with tryptophan (R1070W) in the context of the Wt-CFTR, introduces a bulky group on the face of the coupling helix that interacts with NBD1 and like the substitutions above, this leads to misprocessing. Further support for the hypothesis that this helical segment conferred by ICL4, interacts with the NBD1 surface containing F508 in the full-length protein came from chemical cross-linking studies of engineered interfacial cysteine pairs. Importantly, deletion of F508, impairs chemical cross-linking of the same cysteine pairs in the full-length protein, supporting the idea that this intra-molecular interaction is perturbed in the full-length mutant protein.

Introduction of R1070W or V510D in the F508del-CFTR protein partially corrects folding of the full-length protein, highlighting the idea that even in the absence of F508, assembly of the CFTR can be partially restored through structural changes at key loci in the protein (Thibodeau et al., 2010; Mendoza et al., 2012). Similarly, the second site mutations, previously discussed with regard to their efficacy in stabilizing the isolated F508del-NBD1, i.e., the second site mutations in the ABC conserved core ATP binding subdomains (G550E, R553Q, and R555K) also promote improved processing of the full-length F508del-CFTR. Similarly, second site mutations in unique, flexible regions of NBD1 (i.e., I539T) partially correct the processing defect in F508del-CFTR.

Recent studies by the Lukacs (Rabeh et al., 2012) and the Thomas (Mendoza et al., 2012) groups tested the idea that correction of the thermodynamic and kinetic defects in F508del-NBD1 by second site “stabilizing” mutations may be sufficient to restore proper assembly of the full-length mutant protein. Employing biophysical methods, including circular dichroism, dynamic light scattering, and fluorescence, both groups confirmed that the introduction of “stabilizing mutations” residing in the ABC α-helical subdomain (G550E, R553M, R555K) and the structural diverse region (I539T), fully corrects defects in kinetic and thermal stability of the isolated F508del-NBD1 domain. However, these second site mutations failed to restore folding of the full-length mutant protein to greater than 15% of the Wt-CFTR protein. However, in combination with R1070W, a mutation that reconstitutes a more Wt-like ICL4: NBD1 interface, the NBD1-“stabilizing” mutants mediate full correction and near normal processing. Hence, these authors argue that pharmaceutical interventions which “correct” the thermodynamic instability of NBD1 alone will lack therapeutic efficacy. However, the results of the studies by Riordan and colleagues appear to dispute this view. This group found that compound mutations in the SDR of F508del-NBD1 or deletion of the entire RI region were sufficient to restore Wt folding to the full-length mutant protein in the absence of stabilizing mutations at the ICL4: NBD1 interface (Aleksandrov et al., 2012).

Clearly, there is still much to learn regarding intra-domain and intra-molecular interactions vital for proper folding and assembly of CFTR. The field would benefit greatly from biophysical studies which directly probe the intrinsic determinants for folding or unfolding of the full-length CFTR protein and the major mutant. To date, the only assay for folding of the full-length protein is assessment of the acquisition of complex glycosylation and this readout reflects a complex series of events, with a significant number of these processes being mediated by proteins other than CFTR.

## F508del-CFTR in the Cell

### Defective interaction with the chaperone and ER quality control machinery

CFTR folding is modified by cellular chaperones of the ER which specifically and transiently bind to immature CFTR to prevent aggregation and facilitate efficient folding (Meacham et al., 1999; Wang et al., 2006a; Rosser et al., 2008). These include heat shock protein (Hsp) 70 and its co-chaperone human DnaJ 2 (Hdj-2) which form the cytosolic chaperone complex (Meacham et al., 1999), Hsp90 and its co-chaperone activator of Hsp90 ATPase (Aha1; Wang et al., 2006a), and calnexin (Rosser et al., 2008). Hdj-2/Hsp70 is localized at the cytosolic face of the ER in which Hdj-2 binds to Hsp70 to activate the ATPase activity of the chaperone and binds to specific proteins for folding through its farnesyl tail (Meacham et al., 1999). This complex facilitates both co- and post-translational folding of native CFTR and stabilizes NBD1 as well as its interaction with the R domain (Meacham et al., 1999). The formation and stabilization of the NBD1-R domain interaction then reduces binding and releases the protein from the complex (Meacham et al., 1999). Hsp90 is localized in the cytosol with Aha1 binding to conduct similar roles as Hdj-2 with Hsp70 (Wang et al., 2006a). The mechanism of CFTR folding facilitated by the Aha1/Hsp90 complex remains unknown, however this complex has been shown to be essential for CFTR folding and stability (Wang et al., 2006a). Calnexin, with its lectin domain localized in the ER lumen, also binds to CFTR at two glycosylation sites in extracellular loop 4 of MSD2 (Rosser et al., 2008). The binding of calnexin to CFTR at those sites stabilize MSD2, and facilitates the formation and stabilization of the interaction between MSD2 and MSD1 (Rosser et al., 2008).

The F508del mutation results in altered interactions of CFTR with its cellular chaperones (Meacham et al., 1999; Wang et al., 2006a; Rosser et al., 2008). It has been shown that the Hdj-2/Hsp70 complex interacts with F508del-CFTR approximately twice as much as that of Wt-CFTR (Meacham et al., 1999). This prevents the formation of the NBD1-R domain interaction as the increased residency with the cytosolic chaperone complex buries those sites necessary for that interaction and consequently the folding of the full-length protein (Meacham et al., 1999). In addition, the folding energy required for F508del-CFTR to achieve its native folded conformation far exceeded the capacity of Hsp90 to facilitate its proper folding which emphasizes a major difficulty in correcting the defect of F508del-CFTR (Wang et al., 2006a). There were also more Hsp90 co-chaperones such as Aha1 in the F508del-CFTR interactome which correlates as greater association of the complex with the misfolded protein (Wang et al., 2006a). This increased association blocked the folding pathway of the mutant protein as reduction of Aha1 resulted folding and stability rather than degradation (Wang et al., 2006a). Calnexin was found to interact more with F508del- than Wt-CFTR which also leads to ER retention of the mutant protein (Okiyoneda et al., 2004; Farinha and Amaral, 2005).

The cytosolic chaperone complex can also target the protein for degradation through the ER-associated degradation (ERAD) pathway when correction of misfolding is energetically unfavorable (Younger et al., 2004). ERAD is activated upon the formation the E3 complex by binding of co-chaperones Hdj-2 (Meacham et al., 1999), cytosolic U-box protein CHIP (Meacham et al., 2001), and E2 UbcH5 (Younger et al., 2004) to Hsc or Hsp70 (Zhang et al., 2001). The E3 complex can differentiate between native and misfolded CFTR and ubiquitinates the misfolded protein post-translationally (Younger et al., 2006). The other known ER interacting proteins, including RING domain protein (RMA1/RNF5), E2 ubiquitin-conjugating enzyme (Ubc6e), and the transmembrane quality control factor Derlin-1 further ubiquitinates the misfolded protein (Younger et al., 2006). This complex can detect the aberrant protein at early stages of co-translational folding (i.e., during translation of NBD1, the site of the mutation; Rosser et al., 2008). The ubiquitinated protein will then be targeted to the proteasome for degradation (Younger et al., 2006).

### Altered trafficking and surface stability exhibited by F508del-CFTR

**Figure 2:**
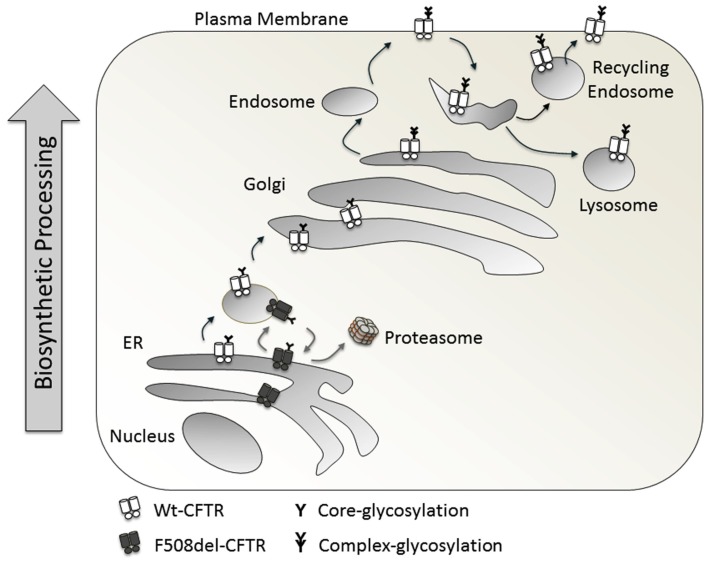
**Biosynthetic processing of Wt-CFTR and F508del-CFTR**.

Normally Wt-CFTR is core glycosylated in the ER and traffics to the Golgi, where it is complex glycosylated (Figure 2). On a Western blot, it runs as two distinct bands: the lower one at around 150 kDa, referred to as Band B (the core glycosylated form) and the heavier Band C (the complex glycosylated form, around 170 kDa). Conventionally, mature CFTR on the cell surface is mostly complex glycosylated (Xie et al., 1996). With the F508del-CFTR mutation, there is a folding defect in the protein which prevents its trafficking from ER to Golgi, represented as a single Band B running at around 150 kDa, and an absence of Band C (Cheng et al., 1990; Qu and Thomas, 1996).

The trafficking of CFTR requires the optimal presence of many chaperones and co-chaperones. Hsps and co-chaperones like Hdj-2 (Meacham et al., 1999), play an important role in folding and trafficking. Also, the coat complex II (COPII) is required for trafficking from the ER. The interaction of a di-acidic ER exit motif within CFTR with COPII is essential for exit from the ER. This interaction is not required for ERAD (Wang et al., 2004). Trafficking of CFTR occurs from the ER to ER-Golgi-intermediate compartment to Golgi. A COPI machinery is described for anterograde and retrograde trafficking between the stacks of Golgi complexes (Yu et al., 2007). Sub-populations of COPI vesicles are described to perform the function of anterograde as well as retrograde transport (Malsam et al., 2005). Prevention of COPI recruitment to the membrane traps Wt-CFTR in the ER. Additionally, prevention of COPI dissociation from the membrane has the same effect. Complete depletion of β-COPI results in trapping of CFTR in the pre-Golgi compartment. The interaction of COPI with CFTR occurs through an RXR motif (i.e., R^553^AR^555^) present on CFTR. F508del-CFTRdoes not traffic to the plasma membrane, but deletion of the RXR motif rescues this phenotype (Zerangue et al., 1999; Kim Chiaw et al., 2009). Thus, the interaction of COPI with F508del-CFTR leads to retrograde transport, back to ER, while COPI interaction with Wt-CFTR leads to anterograde transport. With respect to the cell lines used, the trafficking of CFTR can be COPI dependent or independent. It is COPI dependent in HEK293, HeLa cells, and human epithelial cell lines like HT-29, while COPI independent in BHK and CHO cell lines (Rennolds et al., 2008).

The anterograde transport of CFTR is regulated in part by competitive binding of CFTR with14-3-3 regulatory proteins and COPI. It is considered that increased COPI binding results in retrograde transport, while increased 14-3-3 binding results in anterograde transport. 14-3-3 binds to the RXR motif of CFTR, independent of the phosphorylation status (Liang et al., 2012). An increase in phosphorylated CFTR enhances binding of 14-3-3 with CFTR, and decreases binding of COPI with CFTR. From the perspective of protein biogenesis, cAMP/protein kinase A (PKA) stimulation by forskolin increases CFTR steady-state levels. Additionally, over-expression of 14-3-3 β and ε increases CFTR steady-state levels, thus 14-3-3 seems to protect CFTR from degradation (Liang et al., 2012). Furthermore, considering that proper folding of the protein is important for trafficking, the molecular chaperones, and co-chaperones are therefore important for proper trafficking of CFTR.

#### Cytoskeleton

Despite attempts at correcting the defects of F508del-CFTR, the misfolded protein in cell-based systems continues to result in regulatory and gating channel activity defects (Hwang et al., 1997) which are not apparent in purified protein systems (Li et al., 1993). This major discrepancy indicates the significance of the cellular environment, specifically the intracellular organization, which is absent in purified protein systems, as an essential factor in the regulation and function of the mutant protein (Monterisi et al., 2012). The proteins involved in the intracellular cytoskeletal organization include the Na^+^/H^+^ exchanger regulatory factor isoform protein NHERF2, as well as NHERF1, ezrin, and F-actin which form a complex known as NHERF1-ezrin-actin (Guerra et al., 2005). NHERF proteins are members of the PSD-95/Disk-large/ZO-1 (PDZ) domain protein family which contain two PDZ domains and an ezrin/radixin/moesin (ERM) domain (Hall et al., 1998; Wang et al., 1998). These cytoskeletal proteins interact through their PDZ domains, and bind to the carboxy terminal PDZ motif of CFTR (Hall et al., 1998; Wang et al., 1998). NHERF2 interacts with lysophosphatidic acid 2 (LPA_2_) and CFTR to form the CFTR-NHERF2-LPA_2_ complex which is essential for compartmentalization of cAMP levels, and consequently the regulation of CFTR channel activity (Zhang et al., 2011). NHERF2 is also essential in regulating LPA-mediated phospholipase C-β3 (PLC-β3; Zhang et al., 2011). NHERF1 interacts with the PKA anchoring protein, ezrin, through its ERM domain (Dransfield et al., 1997). Ezrin, in addition to anchoring PKA, connects to the intracellular cytoskeleton by binding with F-actin (Sun et al., 2000). The importance of NHERF1 for the localization and activity of endogenous CFTR has been shown by several groups, such that in human bronchial epithelial (HBE) cells overexpressing NHERF1 the expression levels and activity of CFTR was significantly enhanced (Guerra et al., 2005; Favia et al., 2010; Monterisi et al., 2012). Using HBE cells, previous studies have disrupted these interactions using site-directed mutagenesis, and found that the NHERF1-ezrin-actin complex is essential for stabilizing CFTR by facilitating anterograde trafficking and anchoring to the apical membrane (Moyer et al., 2000; Guerra et al., 2005; Monterisi et al., 2012). Other studies have also claimed that this complex is critical for efficient regulation of CFTR activity and necessary for the localization of sufficient levels of cAMP as well as PKA activity in the appropriate subcortical or membrane compartment (Monterisi et al., 2012).

Based on fluorescence studies, F508del-CFTR has exhibited altered localization in CF bronchial epithelial (CFBE) cells (Guerra et al., 2005; Favia et al., 2010; Monterisi et al., 2012). The cellular localization of NHERF1 differs between CFBE and HBE cells, such that it is expressed in the cytosol and mainly at the cell surface in HBE cells, whereas it was expressed in the cytosol yet absent from the cell surface in CFBE cells (Guerra et al., 2005). The actin cytoskeleton of CFBE cells was also found to be disordered compared to HBE cells (Favia et al., 2010). The differential localization of NHERF1 and the disorganization of the cytoskeleton accounts for the differential localization between F508del-CFTR and Wt-CFTR (Favia et al., 2010). The regulation of F508del-CFTR channel activity was also compromised in CFBE cells (Monterisi et al., 2012). The efficient regulation of CFTR requires cAMP levels and PKA activity to be localized in the subcortical compartment (Monterisi et al., 2012). However, there were significantly higher levels of cAMP and PKA in the cytosol than in the subcortical compartment of CFBE cells compared to HBE cells (Monterisi et al., 2012). Subsequently, it was reported that the disorganization of the actin cytoskeleton of CFBE cells caused these defects, as cAMP could freely diffuse from the subcortical to the cytosolic compartment (Monterisi et al., 2012). Furthermore, since CFTR expression at the cell surface is dependent on the formation of the CFTR-NHERF1-ezrin-actin complex, the disorganization of the intracellular cytoskeleton, resulting from the aforementioned regulatory defects, significantly reduced expression of F508del-CFTR at the apical plasma membrane and led to retention in the ER (Monterisi et al., 2012).

#### GRASP pathway

There is recent evidence that F508del mutant protein can be rescued to the cell surface through an unconventional pathway, referred to as GRASP (Golgi reassembly stacking proteins) dependent secretory pathway. Conventionally the complex glycosylated form of CFTR is considered to be present on the cell surface. But this unconventional Golgi-independent pathway can allow for surface expression of the core glycosylated form of CFTR. The transgenic GRASP55 expression in F508del-CFTR homozygous mouse, could rescue the mutant protein to the plasma membrane and was functional as noted by short-circuit currents using mice colon (Gee et al., 2011). As the mutant F508del-CFTR does have some chloride channel activity, activation of this pathway in the patients having the F508del-CFTR mutation can lead to surface expression of the mutant channel and thereby help in improving function.

#### Lipid rafts

Lipid rafts, small membrane domains which are rich in sphingo-phospholipids and cholesterol, have been implicated to play a role in CF pathology, although the mechanism is controversial. Ceramide is also a key constituent of the lipid raft, and like cholesterol and cholesterol ester, its subcellular distribution is thought to be modified in CF affected tissues (Gentzsch et al., 2007). The PDZ-interacting domain of CFTR is responsible for its localization to lipid rafts within the apical membrane, and facilitates formation of a signaling complex with receptors (Dudez et al., 2008). One such complex with CFTR includes: Tumor necrosis factor receptor 1 (TNFR1) and c-Src, and this complex is thought to play a key role in regulating TNF-α mediated cytokine signaling within the epithelial cell. In CF affected epithelia, ceramide levels and ceramide mediated signaling through lipid raft-localized TNF-α receptors is thought to increase (Dudez et al., 2008; Bodas et al., 2011). The mechanisms underlying the change in ceramide metabolism remain unknown. The proposed role of CFTR in ceramide metabolism via the regulation of endosomal pH has been challenged (Grassmé et al., 2003; Barriere et al., 2009; Haggie and Verkman, 2009). Optimal plasma membrane ceramide concentrations are also regulated by acid-sphingomyelinase (ASM). The balance between ASM and acid ceramidase is essential to maintain optimal ceramide on the cell surface in normal tissues (Teichgräber et al., 2008). CF mice with partial genetic ASM deficiency (*Cftr^−/−^/Smpd1^+/−^*) display reduced inflammation and reduced susceptibility to pseudomonal infection (Grassmé et al., 2008; Teichgräber et al., 2008; Kitatani et al., 2009; Becker et al., 2010; Grassmé et al., 2010). Taken together, this suggests a role for ceramide and lipid raft mediated signaling in CF associated inflammation and pathogenesis in CF mice (Wojewodka et al., 2011). However further studies are required to resolve the current controversies regarding the underlying mechanisms.

#### Recycling and peripheral quality control

The newly formed CFTR has to pass through various quality control check-points at the ER and periphery (post-Golgi). Normally at the periphery, Wt-CFTR undergoes recycling from the plasma membrane to early endosomes and back to the cell surface. Mutant F508del-CFTR is misfolded and is susceptible to ubiquitination, re-routing it from recycling to multivesicular bodies and lysosomal degradation (Sharma et al., 2004). The cellular half-life of Wt-CFTR is greater than 24 h, while that of F508del-CFTR is around 7 h. However, the biochemical half-life of plasma membrane Wt-CFTR is greater than 48 h and that of F508del-CFTR is <4 h (Lukacs et al., 1993; Heda et al., 2001). Thus, even if the mutant F508del-CFTR is rescued by temperature to the plasma membrane, it is less stable, suggesting the role of peripheral quality control in removing the misfolded mutant protein. Molecules involved in clearing the mutant CFTR from the plasma membrane were identified through screens, which revealed the role of chaperones, enzymes, and other molecules involved in ubiquitination like CHIP, Hsc70, and Hsp90. This indicates that similar molecules might be involved in ERAD and post-ER clearance of mutant protein from the plasma membrane (Okiyoneda et al., 2010). The peripheral quality control therefore serves as a check-point for the amount of misfolded protein expressed on the plasma membrane (Wolins et al., 1997).

## Mechanism of Action of Existing Corrector Compounds

Efforts in identification of correctors using HTS approaches have been very fruitful, and there is much we can learn from each CFTR corrector molecule. Hundreds of compounds classified as CFTR correctors have been identified in literature to date (Pedemonte et al., 2005; Van Goor et al., 2006; Carlile et al., 2007; Kalid et al., 2010; Lin et al., 2010; Van Goor et al., 2011). Most of these molecules are deemed unsuitable for clinical use namely due to low efficacy, cell type specificity, and/or toxicity profiles. However, these compounds provide precedent as a useful scientific tool to probe how an ideal corrector may affect CFTR, and a potential scaffold for future drug designs. An understanding of the mechanism of action and binding site of previous generation CFTR correctors would be a leap forward toward the rational drug design of CFTR correctors. To date, no corrector mechanism of action has been entirely resolved, and no clear corrector binding site has been defined. However, through the admirable efforts of many groups, we are now aware of some key features of previous generation correctors.

### Corr-4a

**Figure 3:**
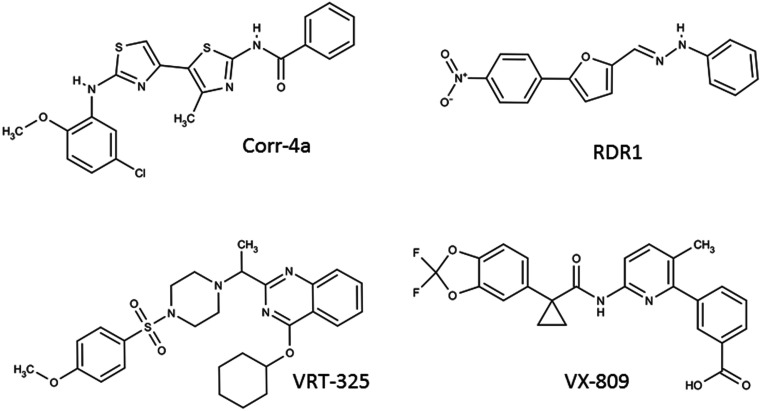
**F508del-CFTR corrector compounds**.

Corr-4a is a bisaminomethylbithiazole derivative identified in a high-throughput screen for CFTR correctors by Pedemonte et al. (2005; Figure 3). Corr-4a has a mild correction effect of the F508del-CFTR (effective in the low μM range), and a nearly complete correction effect on the rare mutant V232D-CFTR (Caldwell et al., 2011). In an effort to understand the mechanism of Corr-4a, Cyr and colleagues examined Corr-4a efficacy in cells with inactivated RMA1 and CHIP ubiquitin ligases. They discovered that Corr-4a affects biogenic intermediates after MSD2 synthesis, and likely only corrects defects in F508del-CFTR which are not recognized by RMA1 E3 ubiquitin ligase (Grove et al., 2009). Additionally, Loo et al. (2009) have demonstrated that treatment with Corr-4a can lead to the partial restoration of inter-domain interactions between MSD1 and MSD2 when F508del-CFTR is expressed in two halves. To date, there is no evidence that Corr-4a acts directly on CFTR. Gene expression profiling studies show that Corr-4a treatment does not significantly alter the cell’s transcriptome, suggesting the effect is fairly specific to CFTR (Sondo et al., 2011), though Corr-4a mediated correction effects have been reported for folding mutants of Pgp and hERG (Van Goor et al., 2011). Since the year of its identification, Corr-4a has proven that pharmacological rescue of CFTR is a viable therapeutic strategy. Corr-4a itself may have low efficacy and an uncertain target, but it has fueled the search for next generation correctors.

### VRT-325

VRT-325, a quinazoline, is a well studied CFTR corrector molecule (Figure 3). It was identified in a HTS designed by Vertex Pharmaceuticals with the support of the Cystic Fibrosis Foundation (Bethesda, USA). VRT-325 is generally effective in cell systems in the 1–10-μM range (Van Goor et al., 2006). Studies by Bear and colleagues have demonstrated that VRT-325 binds directly to purified, reconstituted CFTR, and modifies its ATPase activity. VRT-325 is the first pharmacological chaperone which has been demonstrated to bind directly to CFTR, offering much promise for the future of CFTR correctors as drugs (Kim Chiaw et al., 2010). Despite this finding, VRT-325 has been shown to improve the trafficking of other membrane proteins, including Pgp and hERG folding mutants (Van Goor et al., 2011). Like Corr-4a, VRT-325 does not have a major effect on the cell’s transcriptome, so it is likely producing a protein specific response (Sondo et al., 2011). Many efforts have been put forth to elucidate the mechanism of this correction effect. Limited proteolysis revealed an improvement in the stability of NBD1, but not the second half in the presence of VRT-325 (Yu et al., 2011). The F508del mutation is known to destabilize NBD1, thus restoring its stability is critical step toward restoring F508del-CFTR trafficking and function. Like Corr-4a, VRT-325 has been reported to restore inter-domain interactions at the membrane spanning domains based on co-expressing CFTR in two halves, though this effect was not detectable via limited protease sensitivity of the full-length protein (Loo et al., 2009; Yu et al., 2011). Interestingly, despite its positive effect on CFTR trafficking, in high concentrations VRT-325 (25 μM) was shown to inhibit CFTR-mediated ion flux due to a decrease in ATP-dependent conformational dynamics (Kim Chiaw et al., 2010). This well studied corrector has demonstrated that a small molecule can directly interact with F508del-CFTR, and is capable of partially correcting its intrinsic folding by stabilization of individual domains and inter-domain interfaces.

### RDR1

RDR1 is a CFTR corrector compound first identified in a HTS using isolated NBD1 by Carlile et al. (2007; Figure 3). RDR1 has a mild correction effect compared to equal concentrations of VRT-325, approximately half functional correction at 10 μM (Sampson et al., 2011). Despite its lower efficacy, it represents an interesting scaffold for corrector compounds because it binds directly to isolated NBD1, and is a mild potentiator of CFTR channel activity (Sampson et al., 2011). Differential scanning fluorometry was used to demonstrate that RDR1 improves the thermostability of F508-NBD1. Owing to its direct binding to and stabilization of NBD1, RDR1 is a valuable compound after which future correctors can be designed.

### VX-809

The latest CFTR corrector advancing though clinical trials is VX-809, a novel compound discovered in a HTS by Vertex Pharmaceuticals (Figure 3; Van Goor et al., 2011). Little is known about the mechanism behind VX-809 correction of F508del-CFTR, but current insights are promising. At nanomolar concentrations VX-809 is a highly specific corrector, capable of correcting F508del-CFTR, but not other misfolded membrane proteins (Pgp and hERG mutants). VX-809 confers significant resistance to proteolysis of full-length F508del-CFTR and NBD2 fragments, suggesting an improved overall fold (Van Goor et al., 2011). Further work will be required to understand the molecular interactions which lead to VX-809’s effective and specific F508del-CFTR correction. As a compound only recently made available to the academic community, mechanistic insight is still lacking, however there is much motivation to understand this compound and the potential for more efficacious, and clinically relevant correctors.

## Drug Discovery

As previously described, several structural and functional aberrations require correction in order to restore activity of F508del-CFTR. These structural defects involve intra- and inter-domain interfaces, and subsequently constitute multiple therapeutic targets. Repair of these interfaces can potentially be achieved using pharmacological chaperones (i.e., small molecules) which repair each aberrant site. However, a more desirable therapy would be to identify a single small molecule that intrinsically repairs multiple defects. This would avoid any adverse drug interactions a regimen of multiple therapeutic drugs could incur. It is not enough to repair NBD1 of F508del-CFTR since it has been shown that improved stability of this domain is not sufficient to produce a globally stable protein (Rabeh et al., 2012). Therefore, targeting full-length F508del-CFTR is necessary to restore the biosynthesis, stability, and activity back to wild-type levels.

Although targeting F508del-CFTR directly is desirable, therapeutics which promote trafficking, repress degradation, and increase synthesis of this mutant protein via chaperones and co-chaperones could also be useful. However, this approach would likely have non-specific and toxic effects, since these chaperones are necessary for proper folding of many other proteins. Additionally, it has been shown that even after low temperature rescue, the peripheral protein quality control machinery removes structurally compromised F508del-CFTR from the plasma membrane, and thus could be another target for therapeutics (Lukacs et al., 1993; Heda et al., 2001). Likewise, removal of F508del-CFTR from the cell surface is ubiquitination-dependent and involves several E3 ubiquitin ligases (e.g., CHIP, gp78) which could also be targeted in order to rescue F508del-CFTR (Meacham et al., 2001; Morito et al., 2008).

Interestingly, many solubilizing mutations which enhance the biosynthesis of F508del-CFTR have been identified, and could provide insight into functional interfaces which need to be repaired for restoration of functional activity. Drugs that mimic the structural consequences of these stabilizing mutations could be of therapeutic use. For example, a peptide containing a diarginine (RXR)-based ER retention motif was found to compete with the aberrantly exposed R^553^AR^555^ within NBD1 of F508del-CFTR, subsequently preventing its ER retention and promoting anterograde trafficking to the cell surface (Kim Chiaw et al., 2009). By targeting intracellular pathways which compete with F508del-CFTR biosynthesis, enhanced expression of this major mutant can be functionally rescued to the cell surface.

Another potential route of functional rescue could involve targeting *F508del-CFTR* mRNA. RNA as a drug target has been shown to improve the outcome of type 1 muscular dystrophy *in vitro* (Parkesh et al., 2011; Childs-Disney et al., 2012). Likewise, Bartoszewski et al. (2010) showed that the trinucleotide deletion causing F508del, which is found in the majority of patients with CF (i.e., the out-of-frame *CTT* deletion between amino acids Ile^507^ and Phe^508^) and rendering a synonymous single nucleotide polymorphism at Ile^507^, caused instability of *F508del-CFTR* mRNA due to the enhanced size of hairpin loops relative to wild-type CFTR mRNA. These larger hairpins increased the rate of degradation, and resulted in less mRNA being retained in the cell for translation. In this same study, the authors generated F508del by deleting the trinucleotide corresponding to amino acid Phe^508^ directly (i.e., *TTTdel*) and showed that although this same deletion causes F508del, the RNA primary sequence differed from (*CTTdel)*-*F508del-CFTR* and was sufficient to retain wild-type mRNA loop secondary structure. An abundance of *(TTTdel)-F508del-CFTR* was present at physiological temperature relative to *(CTTdel)-F508del-CFTR*, and allowed for enhanced low temperature rescue at the protein level. This demonstrates the fragility of the naturally occurring *(CTTdel)-F508del-CFTR* mRNA, in addition to the well documented instability at the protein level. Thus, if the loop structure of the naturally occurring F508del-CFTR mRNA could be induced to mimic that of wild-type CFTR (or even *TTTdel*) with therapeutics, sufficient transcript would be available for translation, even though the underlying mutation remains. This approach could enhance the half-life of the misfolded mRNA, increase the synthesis of nascent F508del-CFTR, and establish a novel pool of therapeutic targets which could then be corrected with small molecule protein correctors. Although this approach could improve downstream protein synthesis of F508del-CFTR, it would not directly address the underlying protein folding defects which cause disease.

Although not a small molecule therapeutic, *CFTR* gene therapy, in which the wild-type *CFTR* gene is introduced into the target tissues (e.g., lung, gut), could be another potential approach to treat CF. This delivery method has been under investigation as a CF therapy for over 20 years, and although it may seem straightforward in principle, gene transfer into the lungs has proven to be a problematic endeavor (Griesenbach and Alton, 2012). Gene therapy involves the introduction of foreign DNA using liposomal or viral vectors, and as a result, each approach has had poor clinical outcomes, having issues with low transfer efficiency and immunoreactivity, respectively (Cao et al., 2011). Therefore, a current approach involves pluripotent stem cell therapy using humanamniotic mesenchymal stem cells which are reprogrammed into the required cell type (e.g., bronchial epithelial cells) and which contain wild-type *CFTR* (Paracchini et al., 2012). This method could allow for functional tissue regeneration by means of topical and systemic administration of stem cells, with the goal of replacing dysfunctional tissues containing F508del-CFTR. However, this approach is still in the investigational stage, and favorable experimental results are needed to allow further pursuit at the clinical level.

### Identification of small molecule correctors

There are many chemical libraries which have been compiled by academics and pharmaceutical companies alike in the past few decades, and it is likely that within these libraries an F508del-CFTR corrector or pro-corrector (requiring structural optimization) exists. Therefore, these small molecules need to be included in HTS assays which investigate their ability to functionally correct F508del-CFTR. Three approaches which are used to identify and validate small molecule correctors include:

(1) *In silico* tools to identify putative binding sites for corrector compounds
(2) *In vitro* techniques using purified CFTR protein to identify and validate correctors
(3) Cell-based assays to validate functional correction and investigate mechanism of action of identified small molecules

The choice of chemical compounds to use in HTS, as well as methodologies to investigate and validate novel small molecule correctors will be discussed in detail below.

#### Compound libraries

Compound libraries used in HTS approaches will depend on what is available to the investigator. Most approaches use in house compounds, while others rationally design compounds based on the binding site of the target receptor. The size of the library is an important factor, since the larger the screen the more statistically likely that true positive and thus biological hits will be found. In HTS approaches used to find F508del-CFTR correctors, libraries comprised of thousands to hundreds of thousands of chemical compounds are typically used (Pedemonte et al., 2005; Van Goor et al., 2006; Robert et al., 2010). Structural diversity of compounds in each library is usually large and will subsequently enhance the quality and breadth of the screen, since the likelihood of finding efficacious, specific, and non-toxic correctors *in vivo* will come from identification of drugs which target F508del-CFTR itself, yet do not interfere with normal channel activity.

It is interesting to note that previous corrector screens have used chemical libraries of 2,000–164,000 compounds, and typically the hit rate is ∼0.01–0.03% (Lin et al., 2010). This low yield suggests that larger libraries would be more successful. Furthermore, successful compounds found from HTS must be drug-like, and be able to have therapeutic properties once administered to patients. Thus, any compounds which do not abide by Lipinski’s Rule of Five need to be discarded or optimized at the outset of a screen (Lipinski et al., 2001). Molecules which could become a drug or pro-drug, are retained and tested for corrector activity. Hits from such HTS must then be validated using more rigorous assays of biological activity, usually involving purified CFTR protein. Such leads are then derivatized, optimized, and subjected to further validation.

Lin et al. (2010) used a library containing >3,000 FDA-approved drugs to search for small molecule correctors and potentiators in cell-based assays, and ∼40 chemicals with F508del-CFTR corrector activity were identified. Their choice to screen previously approved drugs is advantageous, since it would streamline application from bench to bedside, saving many years it would normally take to become approved for human indications. Additionally, since CF is a disease in which few therapeutic interventions exist, the Orphan Drug Act allows the approval process to be facilitated, reaching market much sooner than other drugs at the same stage of development (Thorat et al., 2012).

Although not directly addressed by all HTS approaches for correctors, it has been known for many years that F508del-CFTR activity suffers from a channel gating defect (Dalemans et al., 1991). Thus, the consequence of F508del requires more than just a small molecule for trafficking, and so a drug must have potentiator activity as well. Ideally, a small molecule will have both corrector and potentiator activity in order to repair both defects. Thus, corrector-potentiator compounds are needed; one such class of compounds that has shown this activity includes cyanoquinolines (Knapp et al., 2012). Dual screens which address the folding and gating defects would be advantageous in the discovery of a single therapeutic compound.

One approach which is less resource intensive than *in vitro* and *in vivo* studies, yet has had successful applications in identifying bioactive small molecules is that of *in silico* drug discovery (Varady et al., 2003; Klebe et al., 2004; Evers et al., 2005). *In silico* compound libraries can include naturally occurring molecules from flora and fauna, chemicals from *de novo* synthesis, those which do not physically exist but have been computationally designed, and more importantly small molecules which have been rationally designed from protein structures. Since compound structures can be easily modified *in silico*, this approach can be a powerful tool for finding novel therapeutics which satisfy allosteric and electrostatic requirements of the receptor (i.e., F508del-CFTR) binding site(s).

#### *In silico* approaches: virtual screening and rational drug design

*In silico* methods are advantageous since they can identify compounds that bind or “dock” directly to F508del-CFTR, something that cannot be initially confirmed in cell-based assays. Molecular docking has proved useful in the discovery of α1A adrenergic receptor and dopamine D3 receptor antagonists, which is relevant to cardiovascular disease and Parkinson’s disease, respectively (Varady et al., 2003; Evers et al., 2005). In these two cases, the small molecules were found using virtual screening and structure-based rational design from the atomic detail of putative binding sites. Interestingly, many of these designer drugs are inhibitors of their protein targets. Therefore, it may be difficult to design F508del-CFTR correctors which does not inhibit but instead enhance expression and activity.

Virtual screening can be used to identify novel correctors of F508del-CFTR with higher throughput than can be achieved using a cell-based approached (hundreds of millions as opposed to hundreds of thousands). Due to this large volume, and keeping the hit rate constant (based on previous studies), a larger number of correctors will statistically be found. Indeed, reported hit rates for virtual screening are ∼10-fold higher than that for *in vitro* HTS (∼3–5% compared with ∼0.3%; Van Goor et al., 2006; Carlile et al., 2007; Kalid et al., 2010). However, it must be noted that there are currently no crystal structures of CFTR in the presence or absence of small molecules (such structures are desirable for virtual screening), and CFTR structures previously used are homology models based on the related bacterial ABC transporter, Sav1866 (Serohijos et al., 2008; Mornon et al., 2009). Importantly, this type of screening approach is advantageous due to the speed and cost of utility (it is rapid and inexpensive), although a major limitation is that any positive hits need to be confirmed *in vitro*. Furthermore, virtual screening typically uses static or rigid protein structures for docking of small molecules, and so another caveat is that it does not take into account the dynamic nature of proteins.

The purpose of virtual screening is to discover novel scaffolds of small molecule modulators of CFTR activity. This could in turn identify novel therapeutic binding sites within F508del-CFTR which can then be validated *in vitro* and further optimized using quantitative structure-activity relationship studies to create a more efficacious corrector. As such, a study by Kalid et al. (2010) identified several *in silico* correctors which docked to intra-molecular interfaces (e.g., NBD1:NBD2, NBD1:ICL4) within F508del-CFTR, and which were then subsequently validated *in vitro*. This further supports the notion that multiple defects and thus therapeutic targets exist within the mutant protein, and suggests that current Sav1866-based homology models of CFTR must have some degree of accuracy (Serohijos et al., 2008; Mornon et al., 2009).

#### *In vitro*: NBD1 binding assays and techniques using purified protein

Small molecule correctors can also be identified *in vitro*, via binding assays using isolated domains of F508del-CFTR and assays using purified full-length mutant protein. The small molecule RDR1 was found to enhance the thermostability of F508del-NBD1, suggesting that this compound binds directly to improve the folding of this isolated domain (Sampson et al., 2011). From this, RDR1 was extended to the full-length protein in cell surface expression assays, and was also found to improve folding of full-length F508del-CFTR. These studies suggest that this compound repairs an intra-molecular interface involving NBD1 (e.g., NBD1:ICL4, NBD1:NBD2), and facilitates proper folding and subsequent trafficking to the cell membrane, a characteristic of a corrector. However, it is uncertain if this compound is able to potentiate the activity of F508del-CFTR in addition to its corrective properties.

Likewise, purified full-length F508del-CFTR has been used to investigate the effects of small molecule correctors and potentiators. This approach is ideal since it eliminates chaperones as potential targets, and instead identifies F508del-CFTR as the therapeutic receptor. This could allow for faster identification of F508del-CFTR-specific drugs which will not have off target effects and/or toxicities. Although the mechanism of action of known correctors is not well characterized, there have been several studies which suggest that these small molecules bind directly to F508del-CFTR; although at which site within the protein structure is not well understood. For example, Kim Chiaw et al. (2010) demonstrated that VRT-325 binds directly to inhibit the ATPase activity of purified and reconstituted full-length F508del-CFTR, while Yu et al. (2011) showed that VRT-325 decreased the protease susceptibility of F508del-NBD1 in HEK cells, suggesting that this small molecule binds directly to NBD1 or an interface involving NBD1.

### Validation of bioactive compounds: Functional analysis of F508del-CFTR using cell-based systems

#### Cell surface expression and iodide efflux assays

Lead candidate compounds from *in silico* screening, as well as *in vitro* binding and functional assays using purified protein need to be validated in cell-based assays, in order to elucidate the mechanism of action and further improve activity by optimization of chemical structure. One such approach is to use cells overexpressing F508del-CFTR containing a hemagglutinin tag in the fourth extracellular loop, which can be monitored using cell surface immunofluorescence. In this assay, F508del-CFTR which has been “rescued” using correctors will have a hemagglutinin tag exposed to the extracellular matrix, and can subsequently be detected using antibodies. This assay has a reported hit rate of ∼0.06–0.8%, and can be adapted to work with most cell types (Carlile et al., 2007; Robert et al., 2008). However, further validation using biochemical tools is required to assess the mechanism of action of putative direct binding correctors.

Additionally, cell surface expression of F508del-CFTR can be monitored using cells co-expressing a yellow fluorescent protein (YFP) variant which is sensitive to halides (Galietta et al., 2001; Pedemonte et al., 2011b). The fluorescence of this YFP variant is quenched in the presence of chloride or iodide, and can be used to detect CFTR activity and thus cell surface expression. In brief, these F508del-CFTR/YFP expressing cells are put into a solution containing halides, and after addition of forskolin (a CFTR activator) the amount and rate of fluorescence quenching via halide influx is proportional to the amount of functional F508del-CFTR at the cell surface. This method has identified several correctors; however, the mechanism of action of these compounds is poorly understood (Pedemonte et al., 2011b).

Although assays using fluorescence dequenching have also been used to detect corrector activity, they have previously not been amenable to HTS approaches due to the cost and lack of sensitivity. The halide-sensitive fluorophore 6-methoxy-*N*-(3-sulfopropyl) quinolinium is routinely used for this purpose, such that cells expressing F508del-CFTR are loaded with both fluorophore and halide, and after an incubation period, forskolin is added to activate channel activity and the amount of cell surface protein is assessed by means of halide efflux (and an increase in fluorescence; Jayaraman et al., 1999; Mansoura et al., 1999). Academic laboratories have been using this method for many years to assess the activity of CFTR mutants and small molecules on a low throughput scale, however perhaps now this technique can be scaled up to HTS for novel corrector-potentiator compounds since the cost and sensitivity of halide-sensitive fluorescence quenchers and dequenchers has improved in recent years.

Iodide efflux assays using an iodide sensitive electrode have also been one of the main functional assays for assessing F508del-CFTR activity after small molecule “rescue.” In these studies, cells overexpressing F508del-CFTR are loaded with iodide, and after an iodide gradient is establish, forskolin is added to activate CFTR and allow for iodide efflux, which is proportional to the amount of functional protein on the cell surface (Yu et al., 2011). This assay is very sensitive, being able to detect iodide in the nanomolar – micromolar range. However, although these experiments are suitable for investigation of putative mechanisms of action of small molecule correctors and potentiators, this approach is not suitable for HTS assays in its current state, due to the cost of each iodide sensitive probe (multiple probes are required for HTS) as well as the need for calibration prior to each measurement.

## Requirements for the Design of an Ideal Corrector

### Corrector binding site

Little high resolution structural information, other than crystal structures of isolated NBD1, has been published on CFTR (Lewis et al., 2004, 2005). Molecular models based on prokaryotic ABC transporters have been developed to attempt to define the structural features of the protein that allow transduction of the ATP binding and hydrolysis signals in the cytosolic NBDs to increased probability of opening and closing of the conduction pathway through the helical domains that span the membrane (Serohijos et al., 2008; Mornon et al., 2009; Dalton et al., 2012). These models have been instructive in suggesting interactions between NBD1 and NBD2, between the NBDs and the TM domains via the ICLs, and recently a model of the unique regulatory R region and its possible interaction with the remainder of the protein. Ford et al. (2011) have created low resolution structures of CFTR that appear to confirm the close interaction of the TM domains with the NBDs, as predicted from the molecular models (Figure 1).

As described above, the NBDs interact with the TM domains via lengthy helical segments that extend from the TM segments into the cytosol (Figures 1A,B). Shorter helical segments at the foot of the long extensions have been termed “coupling helices” and sit parallel to the NBD surface. There is cross-over between sets of helices in the TM regions such that both TM segments in the first and second half of the protein interact via coupling helices to both NBD domains. The coupling helices may interact with hydrophobic patches on the NBD surface and act as signal transduction platforms to aid in the transfer of information from ATP binding-hydrolysis to channel opening and closing. In fact, the closeness of the F508 residue on the surface of NBD1 to the coupling helix ICL4 suggests that partial disruption of this interaction platform may in part be responsible for the structural and functional consequences of F508del.

As described in the models by the Callebaut group (Mornon et al., 2009), there appears to be subtle but significant movement of domains relative to each other in the channel closed-open transition. The NBDs slide relative to one another upon ATP binding-hydrolysis at the catalytic site and the coupling helices may provide a pivot point, allowing a twisting along the helical extension and TM helices. Kirk and coworkers suggest that changes in the orientation of the long helical segments are important for channel gating and might occur during the ATP binding-hydrolysis cycle (Wang et al., 2010b).

Of particular difficulty in modeling is the unique and intrinsically disordered R domain, which has no structural analog among prokaryotic ABC transporters used to generate CFTR models. The R domain is a highly charged 241 residue linker region possessing multiple phosphorylation sites for PKA and other kinases that joins the two halves of the protein (Tabcharani et al., 1991; Chappe et al., 2005; Kongsuphol et al., 2009). The R domain appears to be a disordered region that is sufficiently flexible to undergo dynamic interactions with other CFTR domains to regulate function. Although the domain is disordered, it contains regions with the propensity to form α-helices in the non-phosphorylated state and upon PKA phosphorylation, this propensity is reduced (Ostedgaard et al., 2000; Baker et al., 2007). The isolated R domain becomes less compact with phosphorylation, and interactions with multiple domains appear to be modulated (Chappe et al., 2005; Baker et al., 2007; Hegedus et al., 2008). The regulatory nature of the R domain suggests it may be a prime binding site for correctors and potentiators whereby the inter-domain interactions are modulated, either strengthening or weakening interactions and inducing altered structural conformations in distant regions of the protein. Modulator interaction at the R domain may correct the domain–domain interactions disrupted through deletion of F508.

Cross-linking experiments have shed some light on conformational maturation in mutant proteins and changes induced by binding of small molecules, and suggest dynamic changes in some regions of the protein. Clarke and co-workers have used a variety of corrector molecules including VRT-325, Corr-4a, and others along with chemical cross-linking and sensitivity to glycosidases to show that small molecule correctors rescue folding mutants such as F508del-CFTR (Wang et al., 2006b, 2007a,b; Loo et al., 2009). They have shown that folding of the TM domains occurs in the absence of the NBDs when treated with corrector VRT-325, indicating that the binding site for this molecule is not in the NBD domains and that direct binding can induce folding of the TMDs. They have also shown that addition of multiple corrector compounds increases the amount of rescued protein, suggesting that these molecules bind to diverse sites in the protein rather than a single corrector site to promote CFTR maturation, likely by multiple mechanisms.

It seems reasonable that corrector binding sites are located at domain–domain interface regions that are critical for signal transduction, and the corrector molecules function by promoting Wt structure and stability at these interfaces to allow the protein to escape the quality control machinery of the ER.

### What’s wrong with first generation correctors?

At least some small molecules, such as butyrate and glycerol, as well as incubation at low temperatures (Denning et al., 1992), can promote increased CFTR trafficking to the cell surface, but these treatments are highly non-specific for CFTR and otherwise not suitable for use in patients. At least some first generation CFTR correctors interact directly with CFTR to promote its rescue (Loo et al., 2009; Wellhauser et al., 2009; Kim Chiaw et al., 2010) rather than exerting their effect non-specifically by increasing total protein expression and lowering fidelity of ER quality control mechanisms. Corrector compounds could work by modifying aberrant interactions of F508del-CFTR with chaperone proteins or degradation pathways (Wang et al., 2006a; Younger et al., 2006).

As described above, most first generation correctors, such as VRT-325, appear to stabilize only some F508del-CFTR domains and domain:domain interactions, and they rescue only a fraction of the protein trafficking to the cell surface (Loo et al., 2005, 2006; Van Goor et al., 2006; Wang et al., 2007b,c; Kim Chiaw et al., 2010). VRT-325, for example, has been estimated to rescue F508del-CFTR to just ∼15% of the maturation efficiency of Wt-CFTR at a concentration of 6.7 μM (Van Goor et al., 2006). CFTR has sufficient affinity for this molecule that it can be used at low micromolar concentrations, promoting maturation of both F508del-CFTR and other processing mutants, and results in some CFTR activity at the cell surface, which are important characteristics of a suitable CFTR corrector molecule for use in patients. However the molecule is not CFTR-specific, rescuing misprocessed Pgp mutants as well as CFTR, meaning it would be expected to have significant off target effects (Wang et al., 2007c).

Most current generation CFTR correctors, such as VX-809 do not potentiate CFTR activity at the cell surface (Van Goor et al., 2011), or indeed many such as VRT-325 partially inhibit channel activity (Kim Chiaw et al., 2010). VRT-325 significantly lowers the ATP hydrolytic function of CFTR at as low as 10 μM concentrations, and at 25 μM reduces the apparent affinity of CFTR for ATP by ∼10-fold, as determined by ATPase activity measurements on purified protein. VRT-325 appears to stabilize NBD1 as it is capable of restoring compactness in this domain, but has no effect on the stability of the C-terminal half of the protein (Yu et al., 2011). It is likely that a small molecule that increases the stability of the NBD domain, but does not bind at this location, will have aberrant and rigid domain–domain interactions with at least the first TM domain of the protein whereby the interactions are not properly modulated by ATP binding-hydrolysis. An ideal CFTR corrector should promote full CFTR maturation without sacrificing regulation and channel function.

VRT-532 is a dual-acting molecule that possesses both weak corrector and robust potentiator activity for mutant CFTR that is not specific to the F508del genotype (Wang et al., 2006b; Wellhauser et al., 2009). However its corrector activity is too low to produce sufficient increases in F508del-CFTR trafficking for meaningful rescue of the protein to the cell surface. This molecule, and others (Mills et al., 2009; Pedemonte et al., 2011a; Phuan et al., 2011; Knapp et al., 2012; Leier et al., 2012), suggest that a multi-acting compound that is capable of both corrector and potentiator roles is possible.

F508del-CFTR that reaches the cell surface has a short half-life at that location (Lukacs et al., 1993; Heda et al., 2001). Many first generation correctors likely do little to stabilize rescued F508del-CFTR at the cell surface. Loss of surface expressed protein is likely to have a major effect on observed channel function at the cell surface over time. Gentzsch et al. (2004) clearly demonstrated that turnover of F508del-CFTR at the cell surface is significantly higher than Wt-CFTR turnover. Under conditions where F508del-CFTR is first rescued by low temperature incubation (27°C), nearly all protein is lost from the cell surface within 4 h incubation at 37°C, while ∼40% of surface expressed Wt-CFTR remains. Collawn and co-workers showed that Corr-4a significantly enhances the stability of rescued F508del-CFTR at the cell surface, up to 12 h at 37°C in surface biotinylation experiments and activity measurements (Varga et al., 2008). While they showed that low temperature treatment reduces proteasomal function, Corr-4a treatment at 37°C may directly inhibit the E1–E3 ubiquitination pathway, as well as reducing endocytosis (Varga et al., 2008). Ussing chamber studies showed that Corr-4a increases cAMP-mediated F508del-CFTR activity by >60% after 6 h incubation at 37°C in the presence of the corrector. Corr-4a treatment alone is therefore not sufficient to produce maximal CFTR activity, however addition of a potentiator molecule can increase the activity of the protein. The off target effects due to the use of a small molecule such as Corr-4a that may alter the ubiquitination pathway and endocytic cell surface protein recycling would preclude such a molecule from being used to treat patients (Varga et al., 2008).

Young et al. (2009) showed that when dynamin-associated removal of Wt-CFTR and temperature-rescued F508del-CFTR from PM is inhibited by the dynamin inhibitor “dynasore,” significantly more CFTR remains at the cell surface, indicating that dynamin-associated processes are important for the cell surface stability of rescued protein. When dynasore is used in conjunction with correction by Corr-4a, significantly more cell surface CFTR is present than either treatment alone, suggesting that multiple mechanisms can be combined to improve CFTR restoration. Stability of surface expression indicates an important mechanism by which CFTR must be corrected to produce sustained robust restoration of CFTR activity.

### Characteristics of an ideal corrector

**Figure 4:**
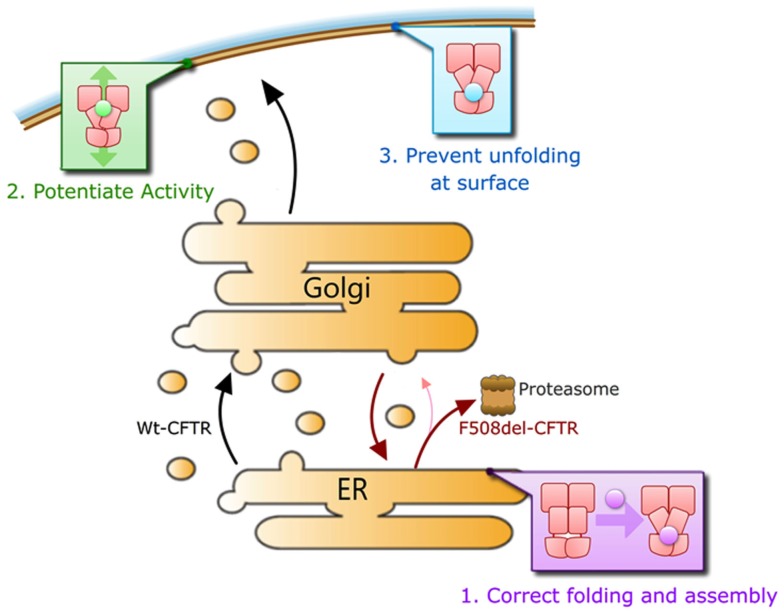
**Overview of molecular defects requiring repair for functional rescue of F508del-CFTR**.

We submit that a corrector will need to repair at least three major defects in CFTR that results from the F508del mutation in order to be maximally effective in correcting the F508del-CFTR phenotype clinically. Namely (1) it must efficiently rescue F508del-CFTR trafficking to the cell surface, ideally to >50% of Wt levels, as heterozygous individuals do not suffer the symptoms of CF. This may involve improving the folding of the protein and/or aiding in escaping ER quality control mechanisms by other means, (2) it must act to increase the compromised activity of F508del-CFTR channels to near Wt levels while retaining phosphorylation-dependent regulation of its activity, and (3) it should increase the stability of the mutant protein on the cell surface to near Wt levels of residence time (Figure 4). A defect in any one of these steps would lead to disease.

Potentiation of the small amounts of F508del-CFTR that naturally reach the cell surface is insufficient to have a measurable improvement in patient clinical outcomes. In F508del-CFTR homozygous individuals, when treated over 16 weeks with the pure CFTR potentiator VX-770 (Ivacaftor; Kalydeco), there was no change in measures of the disease (Flume et al., 2012). This is in contrast to the dramatic improvement of G551D-CFTR patients upon treatment with VX-770 (Ramsey et al., 2011). The G551D-CFTR protein traffics normally to the cell surface and is thought to have a typical Wt residence time, but lacks any CFTR channel function. VX-770 increases the channel open probability of normally trafficked Wt-, G551D-, and F508del-CFTR at the cell surface (Van Goor et al., 2009). Biosynthetic rescue of sufficient amounts of CFTR and normal cell surface residence time are clearly critical to patient clinical response.

As described in other chapters of this Special Topic, correction of significant amounts of F508del-CFTR to the cell surface can be mediated by the pure corrector VX-809 (Van Goor et al., 2011). When patients were treated with this molecule alone in clinical trials, there was little improvement in clinical outcomes over the course of the trial (Clancy et al., 2011). This may be due to a lack of significant function of rescued F508del-CFTR, as VX-809 is a pure corrector molecule (Van Goor et al., 2011). Recent reports suggest that dual treatment of patients with the corrector VX-809 and the potent potentiator VX-770 produces at least some improvement in patient outcomes over the course of the trial (Vertex Pharmaceuticals, 2012), though this improvement does not appear to be to near Wt levels. Full peer-reviewed results of this study are eagerly awaited. It is unclear if VX-809-rescued F508del-CFTR has sufficient residence time at the cell surface. A lack of full Wt-CFTR response under conditions of treatment with both the corrector VX-809 and the potentiator VX-770 suggests the need to address the remaining defect of cell surface residence time in biosynthetically rescued F508del-CFTR.

In our view, correctors should be highly specific for CFTR to avoid off target effects and work via direct binding to the protein to restore proper Wt-like folding. They must correct both inter- and intra-domain folding defects induced by deletion of F508 in NBD1. This would permit proper biosynthetic processing, including typical post-translational modification to that observed in Wt-CFTR, which should result in normal trafficking to the cell surface and proper activity at that location. An F508del-CFTR molecule that has undergone folding close to the conformation seen in Wt would be expected to possess high levels of properly regulated channel activity, and would not be recognized as aberrant protein that is prematurely removed from the cell surface. If a small molecule is not CFTR-specific, not only would it have potentially toxic effects on other proteins and systems that could render them unsuitable for sustained patient use, they could be rapidly removed from the cell via the activity of the Pgp drug pump (Loo et al., 2012).

The term corrector efficacy “ceiling” has been used to describe a theoretical maximal amount of correction that may be afforded to F508del-CFTR, and the concern is whether interventions may be sufficient to restore the activity of the protein to a level that mitigates the most severe clinical symptoms of the disease (Mendoza et al., 2012; Rabeh et al., 2012). It appears that both the folding of NBD1 and its interaction with the remainder of the protein via ICL4 are severely altered when F508 is deleted, resulting in more than one defect that must be corrected. Evidence suggests that correctors that focus on repair of a single one of these defects will be only weakly effective in correcting disease (Mendoza et al., 2012; Rabeh et al., 2012). Perhaps multiple corrector molecules will be required to correct each individual folding defect arising from this mutation. In support of this concept, certain secondary site mutations on the F508del background suppress the F508del mutation (Thibodeau et al., 2010), and other secondary site mutations in conjunction with corrector treatment result in much higher levels of biosynthetic rescue than corrector alone (Yu et al., 2011). There may indeed be a small molecule that can correct these multiple defects, or we may actually reach a ceiling beyond which we cannot further correct CFTR biosynthetic trafficking to the cell surface. In our view the best way to overcome any corrector efficacy ceiling would be to develop a compound that rescues folding as much as possible and simultaneously promotes maximal surface stability of the rescued protein, while it maximizes the regulated channel activity of that surface-targeted protein. The combined effect may be sufficient to overcome CF symptoms. Estimates vary regarding how much CFTR must be rescued (Noone et al., 2000; McKone et al., 2003; Pedemonte et al., 2005) to give normal function, and this would certainly be influenced by the levels of activity of that protein and its residence time at the cell surface, however as heterozygotes are unaffected by disease, a total of 50% restoration of CFTR activity mediated by an aggregate correction-potentiation-surface stabilization mechanism seems to be a desirable target.

Vertex Pharmaceuticals is taking a strategy whereby patients would be treated with two molecules: a pure corrector such as VX-809 to target the protein to the cell surface, and a VX-770, a pure potentiator to increase the activity of the deficient protein at the cell surface. While this strategy appears to be showing promise clinically (Vertex Pharmaceuticals, 2012), we feel the approach is not ideal. Treatment with two molecules gives rise to possible drug interactions and potential increased toxicity issues. It remains to be seen whether VX-809 or VX-770 are sufficient to promote Wt levels of membrane surface stability. Indeed patients may be required to take a third treatment that enhances CFTR cell surface residence time, while not adversely interacting with either of the other two drugs or binding to their binding sites on the protein.

The development of a combined corrector-potentiator-membrane stabilizer molecule would: (1) result in combined repair of all of these defects, which would surpass a “ceiling” for each individual component and greatly improve overall clinical outcomes, (2) reduce drug interactions and toxicity for a combined single treatment versus administration of two to three separate drugs, (3) allow targeting of the single molecule to one target, which would be enhanced over attempts to target three separate drugs to nearby targets on the same protein, and (4) reduce development costs for a single drug versus producing three separate drugs.

### Second generation screens: Better screens will find better compounds

Cell-based screens have been the most successful approach thus far to identify and develop small molecules for the treatment of CFTR mutations (Van Goor et al., 2006, 2009, 2011). These models however typically employ over-expression systems of non-patient derived cells with an endpoint changes in anion conductance or membrane potential as the readout. These systems are anticipated to be highly selective for non-specific compounds, such as molecules that increase protein expression, decrease ER quality control, or even molecules that have direct effects on competing ion channels. Temperature rescuing mutant CFTR followed by acute treatment has been used to identify potentiators while longer treatments with small molecules followed by activity measurements are used to discover corrector molecules. These screens have been effective in identifying potentiators and weak correctors of CFTR trafficking, primarily first generation molecules that may at least partially inhibit CFTR function. Identification of more advanced, second generation small molecules that correct trafficking significantly, potentiate function and maintain cell surface residency will require new methods of screening.

There is significant patient-to-patient variability in disease severity and clinical progression of CF which is not accounted for solely by the associated CFTR genotypic background (Hamosh and Corey, 1993; Li et al., 2011). Gene modifiers are thought to contribute to patient-to-patient variability in disease severity (Wright et al., 2011), and would be anticipated to result in varied response to treatment by small molecules. This variability needs to be taken into account when developing treatments for disease. One can anticipate the future use of patient derived stem cells to produce differentiated lung or other organ cells to test the efficacy of various treatments in a particular patient background before applying the most effective to the patient. Currently, patient derived differentiated cells sourced from explant lung tissue following transplantation are a valuable tool to test the efficacy of small molecules in different genetic backgrounds.

To identify the most effective small molecules with features of correctors, potentiators and small molecules that improve the cell surface stability of mutant CFTR, new approaches will be needed to combine screens for each of these functions. The most clinically useful small molecules will bind directly to mutant CFTR and thus new methods of screening should monitor for direct binding of small molecules to the protein. The subset of small molecules from a library that bind to CFTR with high affinity could then be screened for molecules that correct, potentiate, and enhance surface stability of CFTR in more traditional assays, including evaluation with patient derived differentiated cell systems, where molecules that primarily target quality control machinery or other ion channels would already be selected against in the initial screen.

## Summary and Future Outlook

F508del-CFTR is the most common cystic fibrosis causing mutation, leading to protein misfolding and aberrant trafficking from the ER to Golgi, resulting in a lack of functional expression on the cell surface. As a therapeutic approach, several small molecule correctors have been shown to repair structural defects by binding specifically to F508del-CFTR to improve folding and assembly, and enhance trafficking and expression on the plasma membrane. In addition to these characteristics, such compounds must also stabilize the mutant protein on the cell surface by preventing its unfolding, and further potentiate channel activity. Drug discovery efforts have identified few promising corrector compounds, such as VX-809, which facilitate correction of F508del-CFTR conformation, thereby increasing forward trafficking of the mutant protein; recent clinical trials have also had encouraging results with these therapies. Future therapeutic approaches may require a combination of drugs to repair the aforementioned defects in order to achieve significant clinical outcomes. Alternatively, an ideal pharmacological intervention would involve a single therapeutic small molecule which can correct the structural and functional defects simultaneously.

## Conflict of Interest Statement

The authors declare that the research was conducted in the absence of any commercial or financial relationships that could be construed as a potential conflict of interest.

## Abbreviations

ABC, ATP binding cassette; Aha1, activator of Hsp90 ATPase; ASM, acid-sphingomyelinase; ATP, adenosine triphosphate; cAMP, cyclic adenosine monophosphate; CF, cystic fibrosis; CFTR, cystic fibrosis transmembrane conductance regulator; CHIP, C-terminal Hsp70 interacting protein; COP, coat complex; CoPos, corrector-potentiator compounds; ER, endoplasmic reticulum; ERAD, ER-associated degradation; FRET, fluorescence resonance energy transfer; F508del, deletion of phenylalanine at position 508; GRASP, Golgi reassembly stacking proteins; Hdj-2, human DnaJ 2; Hsp, heat shock protein; HTS, high-throughput screening; ICL, intracellular loop; LPS, lipopolysaccharide; MSD, membrane spanning domain; NMR, nuclear magnetic resonance; NHERF1, Na^+^/H^+^ exchanger regulatory factor isoform 1; NBD, nucleotide binding domain; Pgp, P-glycoprotein; PKA, protein kinase A; PM, plasma membrane; R domain, regulatory domain; TM, transmembrane; Ubc, ubiquitin-conjugating; Wt, wild-type; YFP, yellow fluorescent protein.

## References

[B1] Aleksandrov A. A. Kota P. Aleksandrov L. A. He L. Jensen T. Cui L. Gentzsch M. Dokholyan N. V. Riordan J. R. (2010). Regulatory insertion removal restores maturation, stability and function of DeltaF508 CFTR. J. Mol. Biol. 401, 194–210 10.1016/j.jmb.2010.06.019 20561529 PMC4361937

[B2] Aleksandrov A. A. Kota P. Cui L. Jensen T. Alekseev A. E. Reyes S. He L. Gentzsch M. Aleksandrov L. A. Dokholyan N. V. Riordan J. R. (2012). Allosteric modulation balances thermodynamic stability and restores function of ΔF508 CFTR. J. Mol. Biol. 419, 41–60 10.1016/j.jmb.2012.03.001 22406676 PMC3891843

[B3] Baker J. M. Hudson R. P. Kanelis V. Choy W. Y. Thibodeau P. H. Thomas P. J. Forman-Kay J. D. (2007). CFTR regulatory region interacts with NBD1 predominantly via multiple transient helices. Nat. Struct. Mol. Biol. 14, 738–745 10.1038/nsmb1311 17660831 PMC3943242

[B4] Barriere H. Bagdany M. Bossard F. Okiyoneda T. Wojewodka G. Gruenert D. Radzioch D. Lukacs G. L. (2009). Revisiting the role of cystic fibrosis transmembrane conductance regulator and counterion permeability in the pH regulation of endocytic organelles. Mol. Biol. Cell 20, 3125–3141 10.1091/mbc.E09-01-0061 19420138 PMC2704163

[B5] Bartoszewski R. A. Jablonsky M. Bartoszewska S. Stevenson L. Dai Q. Kappes J. Collawn J. F. Bebok Z. (2010). A synonymous single nucleotide polymorphism in DeltaF508 CFTR alters the secondary structure of the mRNA and the expression of the mutant protein. J. Biol. Chem. 285, 28741–28748 10.1074/jbc.M110.154575 20628052 PMC2937902

[B6] Becker K. A. Riethmüller J. Lüth A. Döring G. Kleuser B. Gulbins E. (2010). Acid sphingomyelinase inhibitors normalize pulmonary ceramide and inflammation in cystic fibrosis. Am. J. Respir. Cell Mol. Biol. 42, 716–724 10.1165/rcmb.2009-0174OC 19635928

[B7] Bodas M. Min T. Mazur S. Vij N. (2011). Critical modifier role of membrane-cystic fibrosis transmembrane conductance regulator-dependent ceramide signaling in lung injury and emphysema. J. Immunol. 186, 602–613 10.4049/jimmunol.1002850 21135173 PMC3119853

[B8] Caldwell R. A. Grove D. E. Houck S. A. Cyr D. M. (2011). Increased folding and channel activity of a rare cystic fibrosis mutant with CFTR modulators. Am. J. Physiol. Lung Cell Mol. Physiol. 301, L346–L352 10.1152/ajplung.00044.2011 21642448 PMC3174745

[B9] Cao H. Yang T. Li X. F. Wu J. Duan C. Coates A. L. Hu J. (2011). Readministration of helper-dependent adenoviral vectors to mouse airway mediated via transient immunosuppression. Gene Ther. 18, 173–181 10.1038/gt.2011.16 20882053

[B10] Carlile G. W. Robert R. Zhang D. Teske K. A. Luo Y. Hanrahan J. W. Thomas D. Y. (2007). Correctors of protein trafficking defects identified by a novel high-throughput screening assay. Chembiochem 8, 1012–1020 10.1002/cbic.200700027 17497613

[B11] Chappe V. Irvine T. Liao J. Evagelidis A. Hanrahan J. W. (2005). Phosphorylation of CFTR by PKA promotes binding of the regulatory domain. EMBO J. 24, 2730–2740 10.1038/sj.emboj.7600747 16001079 PMC1182242

[B12] Cheng S. H. Gregory R. J. Marshall J. Paul S. Souza D. W. White G. A. O’Riordan C. R. Smith A. E. (1990). Defective intracellular transport and processing of CFTR is the molecular basis of most cystic fibrosis. Cell 63, 827–834 10.1016/0092-8674(90)90148-8 1699669

[B13] Childs-Disney J. L. Hoskins J. Rzuczek S. G. Thornton C. A. Disney M. D. (2012). Rationally designed small molecules targeting the RNA that causes myotonic dystrophy type 1 are potently bioactive. ACS Chem. Biol. 7, 856–862 10.1021/cb200408a 22332923 PMC3356481

[B14] Clancy J. P. Rowe S. M. Accurso F. J. Aitken M. L. Amin R. S. Ashlock M. A. Ballmann M. Boyle M. P. Bronsveld I. Campbell P. W. De Boeck K. Donaldson S. H. Dorkin H. L. Dunitz J. M. Durie P. R. Jain M. Leonard A. McCoy K. S. Moss R. B. Pilewski J. M. Rosenbluth D. B. Rubenstein R. C. Schechter M. S. Botfield M. Ordoñez C. L. Spencer-Green G. T. Vernillet L. Wisseh S. Yen K. Konstan M. W. (2011). Results of a phase IIa study of VX-809, an investigational CFTR corrector compound, in subjects with cystic fibrosis homozygous for the F508del-CFTR mutation. Thorax 67, 12–18 10.1136/thoraxjnl-2011-200393 21825083 PMC3746507

[B15] Dalemans W. Barbry P. Champigny G. Jallat S. Dott K. Dreyer D. Crystal R. G. Pavirani A. Lecocq J. P. Lazdunski M. (1991). Altered chloride ion channel kinetics associated with the delta F508 cystic fibrosis mutation. Nature 354, 526–528 10.1038/354526a0 1722027

[B16] Dalton J. Kalid O. Schushan M. Ben-Tal N. Villa-Freixa J. (2012). New model of cystic fibrosis transmembrane conductance regulator proposes active channel-like conformation. J. Chem. Inf. Model 52, 1842–1853 10.1021/ci2005884 22747419

[B17] Denning G. M. Anderson M. P. Amara J. F. Marshall J. Smith A. E. Welsh M. J. (1992). Processing of mutant cystic fibrosis transmembrane conductance regulator is temperature-sensitive. Nature 358, 761–764 10.1038/358761a0 1380673

[B18] Dransfield D. T. Bradford A. J. Smith J. Martin M. Roy C. Mangeat P. H. Goldenring J. R. (1997). Ezrin is a cyclic AMP-dependent protein kinase anchoring protein. EMBO J. 16, 35–43 10.1093/emboj/16.1.35 9009265 PMC1169611

[B19] Du K. Lukacs G. L. (2009). Cooperative assembly and misfolding of CFTR domains in vivo. Mol. Biol. Cell 20, 1903–1915 10.1091/mbc.E08-06-0555 19176754 PMC2663924

[B20] Du K. Sharma M. Lukacs G. L. (2005). The DeltaF508 cystic fibrosis mutation impairs domain-domain interactions and arrests post-translational folding of CFTR. Nat. Struct. Mol. Biol. 12, 17–25 10.1038/nsmb882 15619635

[B21] Dudez T. Borot F. Huang S. Kwak B. R. Bacchetta M. Ollero M. Stanton B. A. Chanson M. (2008). CFTR in a lipid raft-TNFR1 complex modulates gap junctional intercellular communication and IL-8 secretion. Biochim. Biophys. Acta 1783, 779–788 10.1016/j.bbamcr.2008.01.007 18255040 PMC5512004

[B22] Evers A. Hessler G. Matter H. Klabunde T. (2005). Virtual screening of biogenic amine-binding G-protein coupled receptors: comparative evaluation of protein- and ligand-based virtual screening protocols. J. Med. Chem. 48, 5448–5465 10.1021/jm0491804 16107144

[B23] Farinha C. M. Amaral M. D. (2005). Most F508del-CFTR is targeted to degradation at an early folding checkpoint and independently of calnexin. Mol. Cell. Biol. 25, 5242–5252 10.1128/MCB.25.12.5242-5252.2005 15923638 PMC1140594

[B24] Favia M. Guerra L. Fanelli T. Cardone R. A. Monterisi S. Di Sole F. Castellani S. Chen M. Seidler U. Reshkin S. J. Conese M. Casavola V. (2010). Na^+^/H^+^ exchanger regulatory factor 1 overexpression-dependent increase of cytoskeleton organization is fundamental in the rescue of F508del cystic fibrosis transmembrane conductance regulator in human airway CFBE41o- cells. Mol. Biol. Cell 21, 73–86 10.1091/mbc.E09-03-0185 19889841 PMC2801722

[B25] Flume P. A. Liou T. G. Borowitz D. S. Li H. Yen K. Ordoñez C. L. Geller D. E. (2012). Ivacaftor in subjects with cystic fibrosis who are homozygous for the F508del-CFTR mutation. Chest. 10.1378/chest.11-2672 22383668 PMC3435140

[B26] Ford R. C. Birtley J. Rosenberg M. F. Zhang L. (2011). CFTR three-dimensional structure. Methods Mol. Biol. 741, 329–346 10.1007/978-1-61779-117-8_22 21594795

[B27] Galietta L. V. Jayaraman S. Verkman A. S. (2001). Cell-based assay for high-throughput quantitative screening of CFTR chloride transport agonists. Am. J. Physiol. Cell Physiol. 281, C1734–C1742 11600438 10.1152/ajpcell.2001.281.5.C1734

[B28] Gee H. Y. Noh S. H. Tang B. L. Kim K. H. Lee M. G. (2011). Rescue of ΔF508-CFTR trafficking via a GRASP-dependent unconventional secretion pathway. Cell 146, 746–760 10.1016/j.cell.2011.07.021 21884936

[B29] Gentzsch M. Chang X. B. Cui L. Wu Y. Ozols V. V. Choudhury A. Pagano R. E. Riordan J. R. (2004). Endocytic trafficking routes of wild type and DeltaF508 cystic fibrosis transmembrane conductance regulator. Mol. Biol. Cell 15, 2684–2696 10.1091/mbc.E04-03-0176 15075371 PMC420093

[B30] Gentzsch M. Choudhury A. Chang X. B. Pagano R. E. Riordan J. R. (2007). Misassembled mutant DeltaF508 CFTR in the distal secretory pathway alters cellular lipid trafficking. J. Cell. Sci. 120(Pt 3), 447–455 10.1242/jcs.03350 17213331

[B31] Grassmé H. Becker K. A. Zhang Y. Gulbins E. (2008). Ceramide in bacterial infections and cystic fibrosis. Biol. Chem. 389, 1371–1379 10.1515/BC.2008.162 18783339

[B32] Grassmé H. Becker K. A. Zhang Y. Gulbins E. (2010). CFTR-dependent susceptibility of the cystic fibrosis-host to Pseudomonas aeruginosa. Int. J. Med. Microbiol. 300, 578–583 10.1016/j.ijmm.2010.08.011 20951085

[B33] Grassmé H. Jendrossek V. Riehle A. von Kürthy G. Berger J. Schwarz H. Weller M. Kolesnick R. Gulbins E. (2003). Host defense against Pseudomonas aeruginosa requires ceramide-rich membrane rafts. Nat. Med. 9, 322–330 10.1038/nm823 12563314

[B34] Griesenbach U. Alton E. W. (2012). Progress in gene and cell therapy for cystic fibrosis lung disease. Curr. Pharm. Des. 18, 642–662 10.2174/138161212799315993 22229571

[B35] Grove D. E. Rosser M. F. Ren H. Y. Naren A. P. Cyr D. M. (2009). Mechanisms for rescue of correctable folding defects in CFTR Delta F508. Mol. Biol. Cell 20, 4059–4069 10.1091/mbc.E08-09-0929 19625452 PMC2743624

[B36] Guerra L. Fanelli T. Favia M. Riccardi S. M. Busco G. Cardone R. A. Carrabino S. Weinman E. J. Reshkin S. J. Conese M. Casavola V. (2005). Na^+^/H^+^ exchanger regulatory factor isoform 1 overexpression modulates cystic fibrosis transmembrane conductance regulator (CFTR) expression and activity in human airway 16HBE14o- cells and rescues ΔF508 CFTR functional expression in cystic fibrosis cells. J. Biol. Chem. 280, 40925–40933 10.1074/jbc.M505103200 16203733

[B37] Haggie P. M. Verkman A. S. (2009). Defective organellar acidification as a cause of cystic fibrosis lung disease: reexamination of a recurring hypothesis. Am. J. Physiol. Lung Cell Mol. Physiol. 296, L859–L867 10.1152/ajplung.00018.2009 19329540 PMC2692795

[B38] Hall R. A. Ostedgaard L. S. Premont R. T. Blitzer J. T. Rahman N. Welsh M. J. Lefkowitz R. J. (1998). A C-terminal motif found in the β2-adrenergic receptor, P2Y1 receptor and cystic fibrosis transmembrane conductance regulator determines binding to the Na^+^/H^+^ exchanger regulatory factor family of PDZ proteins. Proc. Natl. Acad. Sci. U.S.A. 95, 8496–8501 10.1073/pnas.95.15.8496 9671706 PMC21104

[B39] Hamosh A. Corey M. (1993). Correlation between genotype and phenotype in patients with cystic fibrosis. The Cystic Fibrosis Genotype-Phenotype Consortium. N. Engl. J. Med. 329, 1308–1313 10.1056/NEJM199310283291804 8166795

[B40] Heda G. D. Tanwani M. Marino C. R. (2001). The Delta F508 mutation shortens the biochemical half-life of plasma membrane CFTR in polarized epithelial cells. Am. J. Physiol. Cell Physiol. 280, C166–C174 11121388 10.1152/ajpcell.2001.280.1.C166

[B41] Hegedus T. Serohijos A. W. Dokholyan N. V. He L. Riordan J. R. (2008). Computational studies reveal phosphorylation-dependent changes in the unstructured R domain of CFTR. J. Mol. Biol. 378, 1052–1063 10.1016/j.jmb.2008.03.033 18423665 PMC2556564

[B42] Hwang T. C. Wang F. Yang I. C. Reenstra W. W. (1997). Genistein potentiates wild-type and delta F508-CFTR channel activity. Am. J. Physiol. 273, C988–C998 9316420 10.1152/ajpcell.1997.273.3.C988

[B43] Jayaraman S. Teitler L. Skalski B. Verkman A. S. (1999). Long-wavelength iodide-sensitive fluorescent indicators for measurement of functional CFTR expression in cells. Am. J. Physiol. 277(Pt 1), C1008–C1018 10564094 10.1152/ajpcell.1999.277.5.C1008

[B44] Kalid O. Mense M. Fischman S. Shitrit A. Bihler H. Ben-Zeev E. Schutz N. Pedemonte N. Thomas P. J. Bridges R. J. Wetmore D. R. Marantz Y. Senderowitz H. (2010). Small molecule correctors of F508del-CFTR discovered by structure-based virtual screening. J. Comput. Aided Mol. Des. 24, 971–991 10.1007/s10822-010-9390-0 20976528 PMC4010227

[B45] Kanelis V. Hudson R. P. Thibodeau P. H. Thomas P. J. Forman-Kay J. D. (2010). NMR evidence for differential phosphorylation-dependent interactions in Wt and DeltaF508 CFTR. EMBO J. 29, 263–277 10.1038/emboj.2009.329 19927121 PMC2808376

[B46] Kim Chiaw P. Huan L. J. Gagnon S. Ly D. Sweezey N. Rotin D. Deber C. M. Bear C. E. (2009). Functional rescue of DeltaF508-CFTR by peptides designed to mimic sorting motifs. Chem. Biol. 16, 520–530 10.1016/j.chembiol.2009.04.005 19477416

[B47] Kim Chiaw P. Wellhauser L. Huan L. J. Ramjeesingh M. Bear C. E. (2010). A chemical corrector modifies the channel function of F508del-CFTR. Mol. Pharmacol. 78, 411–418 10.1124/mol.110.065862 20501743

[B48] Kitatani K. Sheldon K. Anelli V. Jenkins R. W. Sun Y. Grabowski G. A. Obeid L. M. Hannun Y. A. (2009). Acid beta-glucosidase 1 counteracts p38delta-dependent induction of interleukin-6: possible role for ceramide as an anti-inflammatory lipid. J. Biol. Chem. 284, 12979–12988 10.1074/jbc.M809500200 19279008 PMC2676030

[B49] Klebe G. Krämer O. Sotriffer C. (2004). Strategies for the design of inhibitors of aldose reductase, an enzyme showing pronounced induced-fit adaptations. Cell. Mol. Life Sci. 61, 783–793 10.1007/s00018-003-3406-z 15095003 PMC11138793

[B50] Knapp J. M. Wood A. B. Phuan P. W. Lodewyk M. W. Tantillo D. J. Verkman A. S. Kurth M. J. (2012). Structure-activity relationships of cyanoquinolines with corrector-potentiator activity in DeltaF508 cystic fibrosis transmembrane conductance regulator protein. J. Med. Chem. 55, 1242–1251 10.1021/jm201372q 22214395 PMC3277286

[B51] Kongsuphol P. Cassidy D. Hieke B. Treharne K. J. Schreiber R. Mehta A. Kunzelmann K. (2009). Mechanistic insight into control of CFTR by AMPK. J. Biol. Chem. 284, 5645–5653 10.1074/jbc.M806780200 19095655 PMC2645823

[B52] Leier G. Bangel-Ruland N. Sobczak K. Knieper Y. Weber W. M. (2012). Sildenafil acts as potentiator and corrector of CFTR but might be not suitable for the treatment of CF lung disease. Cell. Physiol. Biochem. 29, 775–790 22613978 10.1159/000265129

[B53] Lewis H. A. Buchanan S. G. Burley S. K. Conners K. Dickey M. Dorwart M. Fowler R. Gao X. Guggino W. B. Hendrickson W. A. Hunt J. F. Kearins M. C. Lorimer D. Maloney P. C. Post K. W. Rajashankar K. R. Rutter M. E. Sauder J. M. Shriver S. Thibodeau P. H. Thomas P. J. Zhang M. Zhao X. Emtage S. (2004). Structure of nucleotide-binding domain 1 of the cystic fibrosis transmembrane conductance regulator. EMBO J. 23, 282–293 10.1038/sj.emboj.7600040 14685259 PMC1271750

[B54] Lewis H. A. Zhao X. Wang C. Sauder J. M. Rooney I. Noland B. W. Lorimer D. Kearins M. C. Conners K. Condon B. Maloney P. C. Guggino W. B. Hunt J. F. Emtage S. (2005). Impact of the deltaF508 mutation in first nucleotide-binding domain of human cystic fibrosis transmembrane conductance regulator on domain folding and structure. J. Biol. Chem. 280, 1346–1353 10.1074/jbc.M410968200 15528182

[B55] Lewis H. A. Wang C. Zhao X. Hamuro Y. Conners K. Kearins M. C. Lu F. Sauder J. M. Molnar K. S. Coales S. J. Maloney P. C. Guggino W. B. Wetmore D. R. Weber P. C. Hunt J. F. (2010). Structure and dynamics of NBD1 from CFTR characterized using crystallography and hydrogen/deuterium exchange mass spectrometry. J. Mol. Biol. 396, 406–430 10.1016/j.jmb.2009.11.051 19944699

[B56] Li C. Ramjeesingh M. Reyes E. Jensen T. Chang X. Rommens J. M. Bear C. E. (1993). The cystic fibrosis mutation (delta F508) does not influence the chloride channel activity of CFTR. Nat. Genet. 3, 311–316 10.1038/ng0493-311 7526932

[B57] Li W. Sun L. Corey M. Zou F. Lee S. Cojocaru A. L. Taylor C. Blackman S. M. Stephenson A. Sandford A. J. Dorfman R. Drumm M. L. Cutting G. R. Knowles M. R. Durie P. Wright F. A. Strug L. J. (2011). Understanding the population structure of North American patients with cystic fibrosis. Clin. Genet. 79, 136–146 10.1111/j.1399-0004.2010.01502.x 20681990 PMC2995003

[B58] Liang X. Da Paula A. C. Bozóky Z. Zhang H. Bertrand C. A. Peters K. W. Forman-Kay J. D. Frizzell R. A. (2012). Phosphorylation-dependent 14-3-3 protein interactions regulate CFTR biogenesis. Mol. Biol. Cell 23, 996–1009 10.1091/mbc.E11-08-0662 22278744 PMC3302758

[B59] Lin S. Sui J. Cotard S. Fung B. Andersen J. Zhu P. El Messadi N. Lehar J. Lee M. Staunton J. (2010). Identification of synergistic combinations of F508del cystic fibrosis transmembrane conductance regulator (CFTR) modulators. Assay Drug Dev. Technol. 8, 669–684 10.1089/adt.2010.0313 21050065

[B60] Lipinski C. A. Lombardo F. Dominy B. W. Feeney P. J. (2001). Experimental and computational approaches to estimate solubility and permeability in drug discovery and development settings. Adv. Drug Deliv. Rev. 46, 3–26 10.1016/S0169-409X(00)00129-0 11259830

[B61] Loo T. W. Bartlett M. C. Clarke D. M. (2005). Rescue of DeltaF508 and other misprocessed CFTR mutants by a novel quinazoline compound. Mol. Pharm. 2, 407–413 10.1021/mp0500521 16196493

[B62] Loo T. W. Bartlett M. C. Clarke D. M. (2009). Correctors enhance maturation of DeltaF508 CFTR by promoting interactions between the two halves of the molecule. Biochemistry 48, 9882–9890 10.1021/bi9004842 19761259

[B63] Loo T. W. Bartlett M. C. Shi L. Clarke D. M. (2012). Corrector-mediated rescue of misprocessed CFTR mutants can be reduced by the P-glycoprotein drug pump. Biochem. Pharmacol. 83, 345–354 10.1016/j.bcp.2011.11.014 22138447

[B64] Loo T. W. Bartlett M. C. Wang Y. Clarke D. M. (2006). The chemical chaperone CFcor-325 repairs folding defects in the transmembrane domains of CFTR-processing mutants. Biochem. J. 395, 537–542 10.1042/BJ20060013 16417523 PMC1462697

[B65] Lukacs G. L. Chang X. B. Bear C. Kartner N. Mohamed A. Riordan J. R. Grinstein S. (1993). The delta F508 mutation decreases the stability of cystic fibrosis transmembrane conductance regulator in the plasma membrane. Determination of functional half-lives on transfected cells. J. Biol. Chem. 268, 21592–21598 7691813

[B66] Malsam J. Satoh A. Pelletier L. Warren G. (2005). Golgi tethers define subpopulations of COPI vesicles. Science 307, 1095–1098 10.1126/science.1108061 15718469

[B67] Mansoura M. K. Biwersi J. Ashlock M. A. Verkman A. S. (1999). Fluorescent chloride indicators to assess the efficacy of CFTR cDNA delivery. Hum. Gene Ther. 10, 861–875 10.1089/10430349950018274 10223721

[B68] McKone E. F. Emerson S. S. Edwards K. L. Aitken M. L. (2003). Effect of genotype on phenotype and mortality in cystic fibrosis: a retrospective cohort study. Lancet 361, 1671–1676 10.1016/S0140-6736(03)13368-5 12767731

[B69] Meacham G. C. Lu Z. King S. Sorscher E. Toussen A. Cyr D. M. (1999). The Hdj-2/Hsc70 chaperone pair facilitates early steps in CFTR biogenesis. EMBO J. 18, 1492–1505 10.1093/emboj/18.6.1492 10075921 PMC1171238

[B70] Meacham G. C. Patterson C. Zhang W. Younger J. M. Cyr D. M. (2001). The Hsc70 co-chaperone CHIP targets immature CFTR for proteasomal degradation. Nat. Cell Biol. 3, 100–105 10.1038/35070137 11146634

[B71] Mendoza J. L. Schmidt A. Li Q. Nuvaga E. Barrett T. Bridges R. J. Feranchak A. P. Brautigam C. A. Thomas P. J. (2012). Requirements for efficient correction of ΔF508 CFTR revealed by analyses of evolved sequences. Cell 148, 164–174 10.1016/j.cell.2011.11.023 22265409 PMC3266553

[B72] Mills A. D. Yoo C. Butler J. D. Yang B. Verkman A. S. Kurth M. J. (2009). Design and synthesis of a hybrid potentiator-corrector agonist of the cystic fibrosis mutant protein DeltaF508-CFTR. Bioorg. Med. Chem. Lett. 20, 87–91 10.1016/j.bmcl.2009.11.020 19954980 PMC3165007

[B73] Monterisi S. Favia M. Lorenzo G. Cardone R. A. Marzulli D. Reshkin S. J. Casavola V. Zaccolo M. (2012). CFTR regulation in human airway epithelial cells requires integrity of the actin cytoskeleton and compartmentalized cAMP and PKA activity. J. Cell. Sci. 125, 1106–1117 10.1242/jcs.089086 22302988 PMC3324578

[B74] Morito D. Hirao K. Oda Y. Hosokawa N. Tokunaga F. Cyr D. M. Tanaka K. Iwai K. Nagata K. (2008). Gp78 cooperates with RMA1 in endoplasmic reticulum-associated degradation of CFTRDeltaF508. Mol. Biol. Cell 19, 1328–1336 10.1091/mbc.E07-06-0601 18216283 PMC2291415

[B75] Mornon J. P. Lehn P. Callebaut I. (2009). Molecular models of the open and closed states of the whole human CFTR protein. Cell. Mol. Life Sci. 66, 3469–3486 10.1007/s00018-009-0133-0 19707853 PMC11115851

[B76] Moyer B. D. Duhaime M. Shaw C. Denton J. Reynolds D. Karlson K. H. Pfeiffer J. Wang S. Mickle J. E. Milewski M. Cutting G. R. Guggino W. B. Li M. Shanton B. A. (2000). The PDZ-interacting domain of cystic fibrosis transmembrane conductance regulator is required for functional expression in the apical plasma membrane. J. Biol. Chem. 275, 27069–27074 10852925 10.1074/jbc.M004951200

[B77] Noone P. G. Pue C. A. Zhou Z. Friedman K. J. Wakeling E. L. Ganeshananthan M. Simon R. H. Silverman L. M. Knowles M. R. (2000). Lung disease associated with the IVS8 5T allele of the CFTR gene. Am. J. Respir. Crit. Care Med. 162, 1919–1924 11069835 10.1164/ajrccm.162.5.2003160

[B78] Okiyoneda T. Barrière H. Bagdány M. Rabeh W. M. Du K. Höhfeld J. Young J. C. Lukacs G. L. (2010). Peripheral protein quality control removes unfolded CFTR from the plasma membrane. Science 329, 805–810 10.1126/science.1191542 20595578 PMC5026491

[B79] Okiyoneda T. Harada K. Takeya M. Yamahira K. Wada I. Shuto T. Suico M. A. Hashimoto Y. Kai H. (2004). Delta F508 CFTR pool in the endoplasmic reticulum is increased by calnexin overexpression. Mol. Biol. Cell 15, 563–574 10.1091/mbc.E03-06-0379 14595111 PMC329241

[B80] Ostedgaard L. S. Baldursson O. Vermeer D. W. Welsh M. J. Robertson A. D. (2000). A functional R domain from cystic fibrosis transmembrane conductance regulator is predominantly unstructured in solution. Proc. Natl. Acad. Sci. U.S.A. 97, 5657–5662 10.1073/pnas.100588797 10792060 PMC25884

[B81] Paracchini V. Carbone A. Colombo F. Castellani S. Mazzucchelli S. Gioia S. D. Degiorgio D. Seia M. Porretti L. Colombo C. Conese M. (2012). Amniotic mesenchymal stem cells: a new source for hepatocyte-like cells and induction of CFTR expression by coculture with cystic fibrosis airway epithelial cells. J. Biomed. Biotechnol. 2012, 575471 10.1155/2012/575471 22315512 PMC3270433

[B82] Parkesh R. Fountain M. Disney M. D. (2011). NMR spectroscopy and molecular dynamics simulation of r(CCGCUGCGG) reveal a dynamic UU internal loop found in myotonic dystrophy type 1. Biochemistry 50, 599–601 10.1021/bi101896j 21204525 PMC3031998

[B83] Pedemonte N. Diena T. Caci E. Nieddu E. Mazzei M. Ravazzolo R. Zegarra-Moran O. Galietta L. J. (2005). Antihypertensive 1,4-dihydropyridines as correctors of the cystic fibrosis transmembrane conductance regulator channel gating defect caused by cystic fibrosis mutations. Mol. Pharmacol. 68, 1736–1746 16150931 10.1124/mol.105.015149

[B84] Pedemonte N. Tomati V. Sondo E. Caci E. Millo E. Armirotti A. Damonte G. Zegarra-Moran O. Galietta L. J. (2011a). Dual activity of aminoarylthiazoles on the trafficking and gating defects of the cystic fibrosis transmembrane conductance regulator chloride channel caused by cystic fibrosis mutations. J. Biol. Chem. 286, 15215–15226 10.1074/jbc.M110.184267 21383017 PMC3083174

[B85] Pedemonte N. Zegarra-Moran O. Galietta L. J. (2011b). High-throughput screening of libraries of compounds to identify CFTR modulators. Methods Mol. Biol. 741, 13–21 10.1007/978-1-61779-117-8_2 21594775

[B86] Phuan P. W. Yang B. Knapp J. M. Wood A. B. Lukacs G. L. Kurth M. J. Verkman A. S. (2011). Cyanoquinolines with independent corrector and potentiator activities restore DeltaPhe508-cystic fibrosis transmembrane conductance regulator chloride channel function in cystic fibrosis. Mol. Pharmacol. 80, 683–693 10.1124/mol.111.073056 21730204 PMC3187530

[B87] Protasevich I. Yang Z. Wang C. Atwell S. Zhao X. Emtage S. Wetmore D. Hunt J. F. Brouillette C. G. (2010). Thermal unfolding studies show the disease causing F508del mutation in CFTR thermodynamically destabilizes nucleotide-binding domain 1. Protein Sci. 19, 1917–1931 10.1002/pro.479 20687133 PMC2998726

[B88] Qu B. H. Thomas P. J. (1996). Alteration of the cystic fibrosis transmembrane conductance regulator folding pathway. J. Biol. Chem. 271, 7261–7264 10.1074/jbc.271.18.10577 8631737

[B89] Rabeh W. M. Bossard F. Xu H. Okiyoneda T. Bagdany M. Mulvihill C. M. Du K. di Bernardo S. Liu Y. Konermann L. Roldan A. Lukacs G. L. (2012). Correction of both NBD1 energetics and domain interface is required to restore ΔF508 CFTR folding and function. Cell 148, 150–163 10.1016/j.cell.2011.11.024 22265408 PMC3431169

[B90] Ramsey B. W. Davies J. McElvaney N. G. Tullis E. Bell S. C. Dřevínek P. Griese M. McKone E. F. Wainwright C. E. Konstan M. W. Moss R. Ratjen F. Sermet-Gaudelus I. Rowe S. M. Dong Q. Rodriguez S. Yen K. Ordoñez C. Elborn J. S. VX08-770-102 Study Group (2011). A CFTR potentiator in patients with cystic fibrosis and the G551D mutation. N. Engl. J. Med. 365, 1663–1672 10.1056/NEJMoa1105185 22047557 PMC3230303

[B91] Rennolds J. Tower C. Musgrove L. Fan L. Maloney K. Clancy J. P. Kirk K. L. Sztul E. Cormet-Boyaka E. (2008). Cystic fibrosis transmembrane conductance regulator trafficking is mediated by the COPI coat in epithelial cells. J. Biol. Chem. 283, 833–839 10.1074/jbc.M706504200 17932045

[B92] Robert R. Carlile G. W. Liao J. Balghi H. Lesimple P. Liu N. Kus B. Rotin D. Wilke M. de Jonge H. R. Scholte B. J. Thomas D. Y. Hanrahan J. W. (2010). Correction of the Delta phe508 cystic fibrosis transmembrane conductance regulator trafficking defect by the bioavailable compound glafenine. Mol. Pharmacol. 77, 922–930 10.1124/mol.109.062679 20200141

[B93] Robert R. Carlile G. W. Pavel C. Liu N. Anjos S. M. Liao J. Luo Y. Zhang D. Thomas D. Y. Hanrahan J. W. (2008). Structural analog of sildenafil identified as a novel corrector of the F508del-CFTR trafficking defect. Mol. Pharmacol. 73, 478–489 10.1124/mol.107.040725 17975008

[B94] Rosser M. F. Grove D. E. Chen L. Cyr D. M. (2008). Assembly and misassembly of cystic fibrosis transmembrane conductance regulator: Folding defects caused by deletion of F508 occur before and after the calnexin-dependent association of membrane spanning domain (MSD) 1 and MSD2. Mol. Biol. Cell 19, 4570–4579 10.1091/mbc.E08-04-0357 18716059 PMC2575159

[B95] Sampson H. M. Robert R. Liao J. Matthes E. Carlile G. W. Hanrahan J. W. Thomas D. Y. (2011). Identification of a NBD1-binding pharmacological chaperone that corrects the trafficking defect of F508del-CFTR. Chem. Biol. 18, 231–242 10.1016/j.chembiol.2010.11.016 21338920

[B96] Serohijos A. W. Hegedus T. Aleksandrov A. A. He L. Cui L. Dokholyan N. V. Riordan J. R. (2008). Phenylalanine-508 mediates a cytoplasmic-membrane domain contact in the CFTR 3D structure crucial to assembly and channel function. Proc. Natl. Acad. Sci. U.S.A. 105, 3256–3261 10.1073/pnas.0800254105 18305154 PMC2265173

[B97] Sharma M. Pampinella F. Nemes C. Benharouga M. So J. Du K. Bache K. G. Papsin B. Zerangue N. Stenmark H. Lukacs G. L. (2004). Misfolding diverts CFTR from recycling to degradation: quality control at early endosomes. J. Cell Biol. 164, 923–933 10.1083/jcb.200312018 15007060 PMC2172283

[B98] Sondo E. Tomati V. Caci E. Esposito A. I. Pfeffer U. Pedemonte N. Galietta L. J. (2011). Rescue of the mutant CFTR chloride channel by pharmacological correctors and low temperature analyzed by gene expression profiling. Am. J. Physiol. Cell Physiol. 301, C872–C885 10.1152/ajpcell.00507.2010 21753184 PMC3512166

[B99] Sun F. Hug M. J. Lewarchik C. M. Yun C. H. Bradbury N. A. Frizzell R. A. (2000). E3KARP mediates the association of ezrin and protein kinase A with the cystic fibrosis transmembrane conductance regulator in airway cells. J. Biol. Chem. 275, 29539–29546 10.1074/jbc.275.19.14360 10893422

[B100] Tabcharani J. A. Chang X. B. Riordan J. R. Hanrahan J. W. (1991). Phosphorylation-regulated Cl- channel in CHO cells stably expressing the cystic fibrosis gene. Nature 352, 628–631 10.1038/352628a0 1714039

[B101] Teichgräber V. Ulrich M. Endlich N. Riethmüller J. Wilker B. De Oliveira-Munding C. C. van Heeckeren A. M. Barr M. L. von Kürthy G. Schmid K. W. Weller M. Tümmler B. Lang F. Grassme H. Döring G. Gulbins E. (2008). Ceramide accumulation mediates inflammation, cell death and infection susceptibility in cystic fibrosis. Nat. Med. 14, 382–391 10.1038/nm1748 18376404

[B102] Thibodeau P. H. Richardson J. M. III Wang W. Millen L. Watson J. Mendoza J. L. Du K. Fischman S. Senderowitz H. Lukacs G. L. Kirk K. Thomas P. J. (2010). The cystic fibrosis-causing mutation deltaF508 affects multiple steps in cystic fibrosis transmembrane conductance regulator biogenesis. J. Biol. Chem. 285, 35825–35835 10.1074/jbc.M110.131623 20667826 PMC2975206

[B103] Thorat C. Xu K. Freeman S. N. Bonnel R. A. Joseph F. Phillips M. I. Imoisili M. A. (2012). What the Orphan Drug Act has done lately for children with rare diseases: a 10-year analysis. Pediatrics 129, 516–521 10.1542/peds.2011-1798 22371464

[B104] Van Goor F. Hadida S. Grootenhuis P. D. Burton B. Cao D. Neuberger T. Turnbull A. Singh A. Joubran J. Hazlewood A. Zhou J. McCartney J. Arumugam V. Decker C. Yang J. Young C. Olson E. R. Wine J. J. Frizzell R. A. Ashlock M. Negulescu P. (2009). Rescue of CF airway epithelial cell function in vitro by a CFTR potentiator, VX-770. Proc. Natl. Acad. Sci. U.S.A. 106, 18825–18830 10.1073/pnas.0904709106 19846789 PMC2773991

[B105] Van Goor F. Hadida S. Grootenhuis P. D. Burton B. Stack J. H. Straley K. S. Decker C. J. Miller M. McCartney J. Olson E. R. Wine J. J. Frizzell R. A. Ashlock M. Negulescu P. A. (2011). Correction of the F508del-CFTR protein processing defect in vitro by the investigational drug VX-809. Proc. Natl. Acad. Sci. U.S.A. 108, 18843–18848 10.1073/pnas.1105787108 21976485 PMC3219147

[B106] Van Goor F. Straley K. S. Cao D. González J. Hadida S. Hazlewood A. Joubran J. Knapp T. Makings L. R. Miller M. Neuberger T. Olson E. Panchenko V. Rader J. Singh A. Stack J. H. Tung R. Grootenhuis P. D. Negulescu P. (2006). Rescue of DeltaF508-CFTR trafficking and gating in human cystic fibrosis airway primary cultures by small molecules. Am. J. Physiol. Lung Cell Mol. Physiol. 290, L1117–L1130 10.1152/ajplung.00169.2005 16443646

[B107] Varady J. Wu X. Fang X. Min J. Hu Z. Levant B. Wang S. (2003). Molecular modeling of the three-dimensional structure of dopamine 3 (D3) subtype receptor: discovery of novel and potent D3 ligands through a hybrid pharmacophore- and structure-based database searching approach. J. Med. Chem. 46, 4377–4392 10.1021/jm030085p 14521403

[B108] Varga K. Goldstein R. F. Jurkuvenaite A. Chen L. Matalon S. Sorscher E. J. Bebok Z. Collawn J. F. (2008). Enhanced cell-surface stability of rescued DeltaF508 cystic fibrosis transmembrane conductance regulator (CFTR) by pharmacological chaperones. Biochem. J. 410, 555–564 10.1042/BJ20071420 18052931 PMC3939615

[B109] Vertex Pharmaceuticals (2012). Final Data from Phase 2 Combination Study of VX-809 and KALYDECO^™^ (Ivacaftor) Showed Statistically Significant Improvements in Lung Function in People with Cystic Fibrosis Who Have Two Copies of the F508del Mutation. Available at: http://investors.vrtx.com/releasedetail.cfm?ReleaseID = 687394 [Press release].

[B110] Wang C. Protasevich I. Yang Z. Seehausen D. Skalak T. Zhao X. Atwell S. Spencer Emtage J. Wetmore D. R. Brouillette C. G. Hunt J. F. (2010a). Integrated biophysical studies implicate partial unfolding of NBD1 of CFTR in the molecular pathogenesis of F508del cystic fibrosis. Protein Sci. 19, 1932–1947 10.1002/pro.480 20687163 PMC2998727

[B111] Wang W. Wu J. Bernard K. Li G. Wang G. Bevensee M. O. Kirk K. L. (2010b). ATP-independent CFTR channel gating and allosteric modulation by phosphorylation. Proc. Natl. Acad. Sci. U.S.A. 107, 3888–3893 10.1073/pnas.1000264107 20133716 PMC2840504

[B112] Wang S. Raab R. W. Schatz P. J. Guggino W. B. Li M. (1998). Peptide binding consensus of the NHE-RF-PDZ1 domain matches the C-terminal sequence of cystic fibrosis transmembrane conductance regulator (CFTR). FEBS Lett. 427, 103–108 10.1016/S0014-5793(98)00402-5 9613608

[B113] Wang X. Matteson J. An Y. Moyer B. Yoo J. S. Bannykh S. Wilson I. A. Riordan J. R. Balch W. E. (2004). COPII-dependent export of cystic fibrosis transmembrane conductance regulator from the ER uses a di-acidic exit code. J. Cell Biol. 167, 65–74 10.1083/jcb.200408008 15479737 PMC2172508

[B114] Wang X. Venable J. LaPointe P. Hutt D. M. Koulov A. V. Coppinger J. Gurkan C. Kellner W. Matteson J. Plutner H. Riordan J. R. Kelly J. W. Yates J. R. Balch W. E. (2006a). Hsp90 cochaperone Aha1 downregulation rescues misfolding of CFTR in cystic fibrosis. Cell 127, 803–815 10.1016/j.cell.2006.11.023 17110338

[B115] Wang Y. Bartlett M. C. Loo T. W. Clarke D. M. (2006b). Specific rescue of cystic fibrosis transmembrane conductance regulator processing mutants using pharmacological chaperones. Mol. Pharmacol. 70, 297–302 10.1124/mol.106.023986 16624886

[B116] Wang Y. Loo T. W. Bartlett M. C. Clarke D. M. (2007a). Additive effect of multiple pharmacological chaperones on maturation of CFTR processing mutants. Biochem. J. 406, 257–263 10.1042/BJ20070478 17535157 PMC1948964

[B117] Wang Y. Loo T. W. Bartlett M. C. Clarke D. M. (2007b). Correctors promote maturation of cystic fibrosis transmembrane conductance regulator (CFTR)-processing mutants by binding to the protein. J. Biol. Chem. 282, 33247–33251 10.1074/jbc.M703408200 17911111

[B118] Wang Y. Loo T. W. Bartlett M. C. Clarke D. M. (2007c). Modulating the folding of P-glycoprotein and cystic fibrosis transmembrane conductance regulator truncation mutants with pharmacological chaperones. Mol. Pharmacol. 71, 751–758 10.1124/mol.106.030528 17132688

[B119] Wellhauser L. Kim Chiaw P. Pasyk S. Li C. Ramjeesingh M. Bear C. E. (2009). A small-molecule modulator interacts directly with deltaPhe508-CFTR to modify its ATPase activity and conformational stability. Mol. Pharmacol. 75, 1430–1438 10.1124/mol.109.055608 19339490

[B120] Wojewodka G. De Sanctis J. B. Radzioch D. (2011). Ceramide in cystic fibrosis: a potential new target for therapeutic intervention. J. Lipids 2011, 674968 21490807 10.1155/2011/674968 PMC3066841

[B121] Wolins N. Bosshart H. Küster H. Bonifacino J. S. (1997). Aggregation as a determinant of protein fate in post-Golgi compartments: role of the luminal domain of furin in lysosomal targeting. J. Cell Biol. 139, 1735–1745 10.1083/jcb.139.7.1735 9412468 PMC2132652

[B122] Wright F. A. Strug L. J. Doshi V. K. Commander C. W. Blackman S. M. Sun L. Berthiaume Y. Cutler D. Cojocaru A. Collaco J. M. Corey M. Dorfman R. Goddard K. Green D. Kent J. W. Jr. Lange E. M. Lee S. Li W. Luo J. Mayhew G. M. Naughton K. M. Pace R. G. Pare P. Rommens J. M. Sandford A. Stonebraker J. R. Sun W. Taylor C. Vanscoy L. L. Zou F. Blangero J. Zielenski J. O’Neal W. K. Drumm M. L. Durie P. R. Knowles M. R. Cutting G. R. (2011). Genome-wide association and linkage identify modifier loci of lung disease severity in cystic fibrosis at 11p13 and 20q13.2. Nat. Genet. 43, 539–546 10.1038/ng.838 21602797 PMC3296486

[B123] Xie J. Drumm M. L. Zhao J. Ma J. Davis P. B. (1996). Human epithelial cystic fibrosis transmembrane conductance regulator without exon 5 maintains partial chloride channel function in intracellular membranes. Biophys. J. 71, 3148–3156 10.1016/S0006-3495(96)79508-5 8968585 PMC1233803

[B124] Young A. Gentzsch M. Abban C. Y. Jia Y. Meneses P. I. Bridges R. J. Bradbury N. A. (2009). Dynasore inhibits removal of wild-type and DeltaF508 cystic fibrosis transmembrane conductance regulator (CFTR) from the plasma membrane. Biochem. J. 421, 377–385 10.1042/BJ20090389 19442237

[B125] Younger J. M. Chen L. Ren H. Y. Rosser M. F. Turnbull E. L. Fan C. Y. Patterson C. Cyr D. M. (2006). Sequential quality-control checkpoints triage misfolded cystic fibrosis transmembrane conductance regulator. Cell 126, 571–582 10.1016/j.cell.2006.06.041 16901789

[B126] Younger J. M. Ren H. Y. Chen L. Fan C. Y. Fields A. Patterson C. Cyr D. M. (2004). A foldable CFTR{Delta}F508 biogenic intermediate accumulates upon inhibition of the Hsc70-CHIP E3 ubiquitin ligase. J. Cell Biol. 167, 1075–1085 10.1083/jcb.200410065 15611333 PMC2172621

[B127] Yu W. Chiaw P. K. Bear C. E. (2011). Probing conformational rescue induced by a chemical corrector of F508del-cystic fibrosis transmembrane conductance regulator (CFTR) mutant. J. Biol. Chem. 286, 24714–24725 10.1074/jbc.M110.185124 21602569 PMC3137047

[B128] Yu Y. Platoshyn O. Safrina O. Tsigelny I. Yuan J. X. Keller S. H. (2007). Cystic fibrosis transmembrane conductance regulator (CFTR) functionality is dependent on coatomer protein I (COPI). Biol. Cell 99, 433–444 10.1042/BC20060114 17388782

[B129] Zerangue N. Schwappach B. Jan Y. N. Jan L. Y. (1999). A new ER trafficking signal regulates the subunit stoichiometry of plasma membrane KATP channels. Neuron 22, 537–548 10.1016/S0896-6273(00)80708-4 10197533

[B130] Zhang W. Penmatsa H. Ren A. Punchihewa C. Lemoff A. Yan B. Fujii N. Naren A. P. (2011). Functional regulation of cystic fibrosis transmembrane conductance regulator-containing macromolecular complexes: a small-molecule inhibitor approach. Biochem. J. 435, 451–462 10.1042/BJ20101725 21299497 PMC3177239

[B131] Zhang Y. Nijbroek G. Sullivan M. L. McCracken A. A. Watkins S. C. Michaelis S. Brodsky J. L. (2001). Hsp70 molecular chaperone facilitates endoplasmic reticulum-associated protein degradation of cystic fibrosis transmembrane conductance regulator in yeast. Mol. Biol. Cell 12, 1303–1314 11359923 10.1091/mbc.12.5.1303 PMC34585

